# A novel, non-spirometric “BMP index” for predicting severe adverse outcomes in fibrosing interstitial lung diseases

**DOI:** 10.1038/s41598-025-32619-1

**Published:** 2025-12-14

**Authors:** Tang-Hsiu Huang, Hsin-Yu Hou, Han-Yu Chang, Chia-Hao Hu, Hung-I. Kuo, Hong-Ping Er, Yu-Wei Wu, Chien-Yu Lin, Yau-Lin Tseng, Ju-Ming Wang, Li-Ting Huang, Chia-Tse Weng, Sheng-Hsiang Lin, Chi-Chang Shieh, Chao-Liang Wu

**Affiliations:** 1 Institute of Clinical Medicine, College of Medicine, National Cheng Kung University, Tainan, Taiwan; 2 Division of Chest Medicine, Department of Internal Medicine, National Cheng Kung University Hospital, College of Medicine, National Cheng Kung University, Tainan, Taiwan; 3 Division of Chest Medicine, Department of Internal Medicine, Tainan Municipal Hospital, Tainan, Taiwan; 4 Chest Hospital, Ministry of Health and Welfare, Tainan, Taiwan; 5 Division of Chest Medicine, Medical Department, Tainan Hospital, Ministry of Health and Welfare, Tainan, Taiwan; 6 Division of Thoracic Surgery, Department of Surgery, National Cheng Kung University Hospital, College of Medicine, National Cheng Kung University, Tainan, Taiwan; 7 Department of Biotechnology and Bioindustry Sciences, College of Bioscience and Biotechnology, National Cheng Kung University, Tainan, Taiwan; 8 International Research Center for Wound Repair and Regeneration, National Cheng Kung University, Tainan, Taiwan; 9 Department of Medical Imaging, National Cheng Kung University Hospital, College of Medicine, National Cheng Kung University, Tainan, Taiwan; 10 Division of Allergy, Immunology and Rheumatology, Department of Internal Medicine, National Cheng Kung University Hospital, College of Medicine, National Cheng Kung University, Tainan, Taiwan; 11 Department of Public Health, College of Medicine, National Cheng Kung University, Tainan, Taiwan; 12 Biostatistics Consulting Center, National Cheng Kung University Hospital, College of Medicine, National Cheng Kung University, Tainan, Taiwan; 13 Department of Pediatrics, National Cheng Kung University Hospital, College of Medicine, National Cheng Kung University, Tainan, Taiwan; 14 Department of Biochemistry and Molecular Biology, College of Medicine, National Cheng Kung University, Tainan, Taiwan; 15 Ditmanson Medical Foundation Chia-Yi Christian Hospital, Chiayi, Taiwan

**Keywords:** Body mass index, Mucin-1, Pentraxin 3, Pulmonary fibrosis, Acute exacerbation, Mortality, Biomarkers, Diseases, Medical research, Risk factors

## Abstract

Fibrosing interstitial lung diseases (fILDs) exhibit diverse clinical courses and prognoses, with no established biomarkers for predicting acute exacerbation (AE) and early death. This study introduces a novel composite “BMP index”, integrating body mass index (BMI), mucin-1, and pentraxin 3 (PTX3) to assess AE and early death risk in fILDs. Between January, 2017, and May, 2025, we enrolled 366 patients with fILDs diagnosed, managed and followed at three hospitals in southern Taiwan. Using stratified randomization with a ratio 3:2, participants were assigned to derivation (218 patients) and validation (148 patients) cohorts. Baseline circulating mucin-1 and PTX3 levels were measured via enzyme-linked immunosorbent assay. One point was assigned for each of the three components of the BMP index: BMI < 24 kg/m^2^, mucin-1 ≥ 2.5 ng/mL, and PTX3 ≥ 2.2 ng/mL, yielding a total score from 0 to 3. The BMP index consistently stratified risk across the derivation, validation, and combined whole cohorts, with higher scores indicating increased AE and early death risks. Sensitivity analyses and comparative performance assessments confirming its robustness and superior predictive performance compared to multiple recently reported predictors. Our findings support the BMP index as a non-spirometric, noninvasive, radiation-free tool for early clinical risk stratification in fILDs.

**Supplementary Information:**

The online version contains supplementary material available at 10.1038/s41598-025-32619-1.

## Introduction

Interstitial lung diseases (ILDs) encompass a large group of idiopathic and secondary pathologic lung conditions. Regardless of etiologies and initial patterns, certain ILDs may lead to irreversible fibrotic destruction of lung parenchyma, evolving into fibrosing ILDs (fILDs)^1–3^. There is heterogeneity in trajectories and prognosis among patients with fILDs. Currently, there are no well-established biomarkers for predicting the risks of acute exacerbation (AE) and death, even in patients with the most severe subtypes of fILDs, such as idiopathic pulmonary fibrosis (IPF) and progressive pulmonary fibrosis (PPF)^3–8^.

Pulmonary function tests and high-resolution computed tomography (HRCT) are essential parts in the evaluation of fILDs. Poor pulmonary function and honeycombing on HRCT at baseline have been reported as possible predictors of subsequent progressive fibrosis, AE, and decreased survival^1,5–9^. However, because fILDs are clinically characterized by cough and dyspnea, obtaining accurate and reproducible measurements of spirometric volumes and diffusion capacity for carbon monoxide (D_LCO_) can be challenging in practice, as these symptoms often interfere with testing performance^3,4^. While honeycombing is characteristic of the most fibrotic “usual interstitial pneumonia” (UIP) pattern, fILDs can also manifest as non-UIP patterns^1,3,6,10,11^. AE may occur in patients with fILDs who exhibit only mild respiratory impairment and no honeycombing^8,9^.

Fibrosing ILDs can lead to significant weight loss^12–14^. Body mass index (BMI), a widely recognized nutritional metric^12^, has been linked to adverse outcomes in patients with fILDs, particularly IPF^12–20^. While underweight patients tend to have the poorest prognosis, even those with a normal BMI may experience worse outcomes compared to individuals who are overweight or obese^13–16,19^. Moreover, several circulating macromolecules have emerged as potential blood biomarker candidates for the prognosis of fILDs^21,22^. Mucin-1, also known for its epitopic derivatives such as Krebs von den Lungen-6 (KL-6) and CA15-3, is a transmembrane O-glycoprotein expressed by type II alveolar cells^23,24^. Pentraxin 3 (PTX3), on the other hand, is an acute-phase pattern-recognition molecule of the long-pentraxin family produced by multiple cell types^25^. Both mucin-1 and PTX3 are involved in crucial cellular functions and have been implicated in fibrogenesis^23–25^, with their blood levels elevated in fILDs. Previous studies have shown correlations between blood mucin-1/KL-6/CA15-3 levels and the severity of respiratory impairment, as well as AE risks, in various ILDs^22–24^. Recently, we reported the utility of plasma mucin-1 levels, with a cutoff value of 2.5 ng/mL, in predicting risks of AE and death in two distinct cohorts of patients with IPF receiving different antifibrotics^26,27^. We also found that baseline plasma PTX3 levels, with a cutoff value of 2.2 ng/mL, predicted risks of AE and death in patients with fILDs^25^. Nevertheless, to date, neither macromolecule has been definitely accepted as prognostic biomarkers for fILDs.

In this study, we aimed to introduce a novel composite “BMP index” for predicting the risks of AE and early death in patients with fILDs. The BMP index mitigates the limitations of spirometry and D_LCO_, and is noninvasive and radiation-free. It incorporates three surrogate indicators of different pathogenetic aspects of fILDs. The “B” component represents the baseline BMI, indicating the systemic catabolic effect of fILDs. The “M” component represents baseline plasma mucin-1 levels, indicating alveolar fibrotic destruction. The “P” component is determined by baseline plasma PTX3 levels, indicating alveolar injury.

## Results

**Fig. 1:**
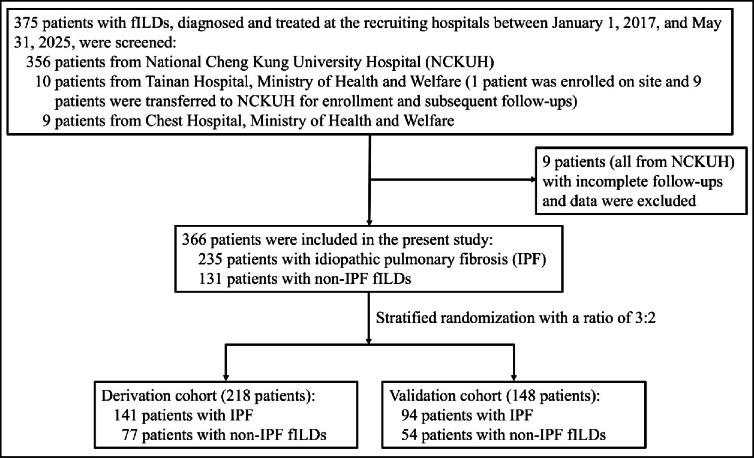
The inclusion and exclusion flowchart of this study.

We enrolled a total of 366 patients, including 347 from NCKUH, 10 from TH (of whom 1 was enrolled on site and 9 were transferred to NCKUH for enrollment and subsequent management), and 9 from CH (Fig. 1). The median age of the whole cohort was 71.9 years (IQR 64.4–79.2), with 270 (74%) being male. Overall, the median follow-up duration was 117 weeks (IQR 56–239). All patients were followed for at least 6 months, and 301 (82%) for at least 2 years, or until death. Specific fILD diagnoses of the patients are listed in Supplementary Table S1. A total of 111 patients (all having non-IPF fILDs) had initiated immunosuppressive therapy prior to study enrollment, whereas 183 patients subsequently received antifibrotic treatment during the study period. After stratified randomization, 218 patients were assigned to the derivation cohort and 148 to the validation cohort. There were no significant differences between the two cohorts in terms of characteristics, treatments, and outcomes (Table 1).

**Table 1:** Baseline characteristics and outcomes of the derivation and validation cohorts.

Baseline characteristics and outcome events	Derivation cohort (*n* = 218)	Validation cohort (*n* = 148)
Age, years	71.6 (63.8–78.7)	72.8 (65.6–80.2)
Sex female, n (%)	56 (26)	40 (27)
Male, n (%)	162 (74)	108 (73)
Body mass index, kg/m^2^	23.7 ± 3.8	23.6 ± 3.9
Cigarette smoking status never smoker, n (%)	103 (47)	65 (44)
Current smoker, n (%)	19 (9)	15 (10)
Former smoker, n (%)	96 (44)	68 (46)
Charlson comorbidity index	5 (3–7)	5 (3–6)
Pulmonary hypertension probability^1^ low, n (%)	139 (64)	94 (61)
Interm., n (%)	56 (26)	32 (22)
High, n (%)	23 (10)	25 (17)
Diagnosis IPF	141 (64)	94 (64)
Non-IPF fILDs	77 (36)	54 (36)
FVC, L	2.19 ± 0.75	2.24 ± 0.87
FVC, % prediction	72.4 ± 20.0	75.1 ± 24.1
D_LCO_, mL/min/mmHg	9.5 (7.1–13.2)	10.2 (7.4–13.7)
D_LCO_, % prediction	59 (43–77)	63 (49–84)
Mucin-1 level, ng/mL	0.97 (0.51–2.40)	1.25 (0.57–2.89)
Pentraxin 3 level, ng/mL	1.41 (0.89–2.69)	1.53 (0.89–2.64)
Stages based on the GAP index stage 1, n (%)	98 (45)	66 (45)
Stage 2, n (%)	77 (35)	51 (34)
Stage 3, n (%)	43 (20)	31 (21)
BMP index 0, n (%)	75 (34)	50 (34)
1, n (%)	69 (32)	49 (33)
2, n (%)	50 (23)	28 (19)
3, n (%)	24 (11)	21 (14)
Duration of follow-ups, weeks	124 (64–242)	102 (47–222)
Already initiated immunosuppressants, n (%)^2^	68 (31)	43 (29)
Received antifibrotics during the study period, n (%)	112 (51)	71 (48)
Nintedanib, n (%)	60 (28)	29 (20)
Pirfenidone, n (%)	35 (16)	34 (23)
Ever switched between the two agents, n (%)^3^	17 (7)	8 (5)
Acute exacerbation, n (%)	60 (28)	48 (32)
Early death, n (%)	55 (25)	47 (31)

Categorical data are presented as counts and percentages, and continuous variables are presented as means (± standard deviation) if normally distributed or medians (interquartile range) if non-normally distributed. There were no significant differences between the 2 cohorts in terms of all characteristics and outcomes. ^1^The classification is based on 2022 ESC/ERS Guidelines for the diagnosis and treatment of pulmonary hypertension^44^. ^2^Immunosuppressants (referring to systemic corticosteroids and non-steroid immunosuppressants) were administered to patients with non-IPF fILDs. ^3^These patients transitioned between the two antifibrotic agents due to intolerance to adverse effects. BMP, body mass index, mucin-1, pentraxin 3; D_LCO_, diffusion capacity for carbon monoxide; fILD, fibrosing interstitial lung diseases; FVC, forced vital capacity; GAP, gender-age-physiology; Interm., intermediate; IPF, idiopathic pulmonary fibrosis.

### BMP index and AE

**Fig. 2:**
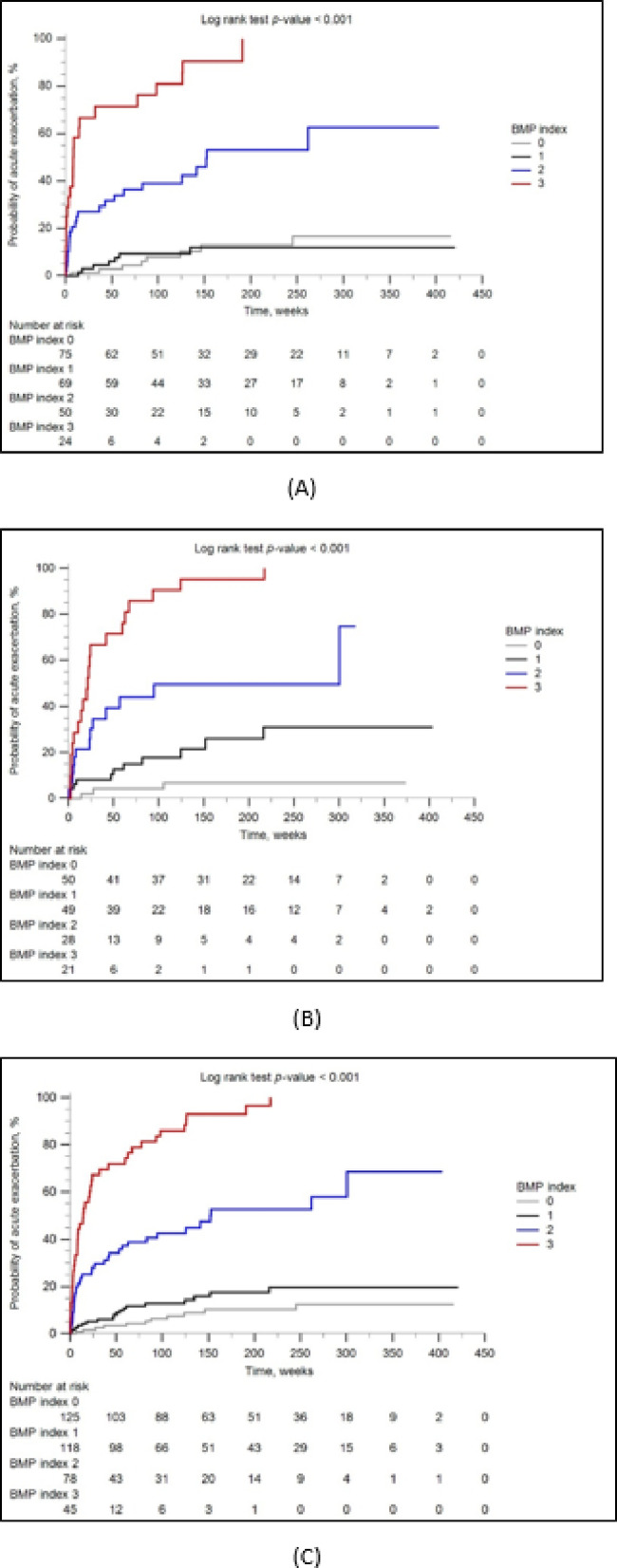
Kaplan–Meier curves illustrating the cumulative probability of developing acute exacerbation in the derivation cohort (**A**), validation cohort (**B**), and the combined whole cohort (**C**).

During the study period, 108 patients (30%) experienced at least one episode of AE (60 patients in the derivation and 48 in the validation cohort). The median time between study enrollment and the first AE was 21.5 weeks (IQR 4.8–72.3), and 89 patients (82%) developed first episode of AE within 2 years of enrollment (47 patients in the derivation and 42 in the validation cohort). In both cohorts, patients who developed AE were more likely to have, at baseline, a lower BMI, higher CCI, higher levels of mucin-1 and PTX3, higher probabilities of PH, poorer pulmonary functions, higher GAP stages, and higher BMP indices compared to patients without AE (Supplementary Table S2). Higher BMP indices indicated greater proportions of patients experiencing AE during follow-ups in both cohorts (Supplementary Table S3), irrespective of immunosuppressant use before study enrollment (Supplementary Table S4) or subsequent administration of antifibrotic therapy (Supplementary Table S5). In Kaplan–Meier analyses, patients in the derivation cohort with BMP indices ≥ 2 had higher cumulative probabilities of AE during follow-up, with index 3 associated with even higher probabilities than index 2. This positive correlation with AE probabilities was not prominent for indices 0 and 1 in the derivation cohort. Nevertheless, in the validation cohort and the combined whole cohort, the BMP index effectively stratified the probabilities of AE starting from index 1, indicating that patients with consecutively higher BMP indices had higher cumulative probabilities of AE (Fig. 2A,B,C). Univariate and multivariable Fine-Gray regression analyses yielded concordant results: starting from index 2 in the derivation cohort and from index 1 in the validation and whole cohorts, increasing BMP indices indicated consecutively higher subdistribution hazard ratios of AE (Table 2). When the analysis was restricted to patients who developed AE within two years of enrollment, the BMP index continued to demonstrate effective stratification of AE risk (Supplementary Fig. S1A–C and Supplementary Table S6).

**Table 2:** Univariate and multivariable Fine-Gray subdistribution regression analyses of the risk of acute exacerbation in patients across the derivation, validation, and combined whole cohorts.

BMP index	Derivation cohort (*n* = 218)		Validation cohort (*n* = 148)		Whole cohort (*n* = 366)	
	Crude sdHR of AE^1^ (95% CI)	*P* value	Crude sdHR of AE^1^ (95% CI)	*P* value	Crude sdHR of AE^1^ (95% CI)	*P* value
0	Ref.	Ref.	Ref.	Ref.	Ref.	Ref.
1	0.90 (0.33–2.46)	0.84	4.01 (1.12–14.30)	0.032	1.74 (0.83–3.65)	0.15
2	5.31 (2.42–11.67)	< 0.001	9.99 (2.82–35.40)	< 0.001	6.61 (3.38–12.94)	< 0.001
3	19.76 (9.05–43.15)	< 0.001	40.59 (12.49–131.90)	< 0.001	25.73 (13.71–48.30)	< 0.001

BMP index	Adjusted sdHR of AE^2^ (95% CI)	*P* value	Adjusted sdHR of AE^2^ (95% CI)	*P* value	Adjusted sdHR of AE^2^ (95% CI)	*P* value
0	Ref.	Ref.	Ref.	Ref.	Ref.	Ref.
1	0.71 (0.25–2.06)	0.53	2.76 (0.77–9.90)	0.12	1.40 (0.66–2.99)	0.38
2	4.22 (1.90–9.36)	<0.001	5.04 (1.34–18.99)	0.017	4.63 (2.32–9.25)	< 0.001
3	11.86 (5.32–26.43)	<0.001	18.73 (5.45–64.43)	< 0.001	13.80 (7.08–26.88)	< 0.001

^1^Values were derived from univariate Fine-Gray subdistribution hazard regression analysis controlling for the competing risk of death.

^2^Values were derived from multivariable Fine-Gray subdistribution hazard regression analysis, which accounted for the competing risk of death and also adjusted for GAP stages, the Charlson comorbidity index, and echocardiographic probabilities of pulmonary hypertension. AE, acute exacerbation; GAP, gender-age-physiology; Ref., the reference group in the determination of subdistribution hazard ratios; sdHR, subdistribution hazard ratio; 95% CI, 95% confidence interval.

### BMP index and early death

**Fig. 3:**
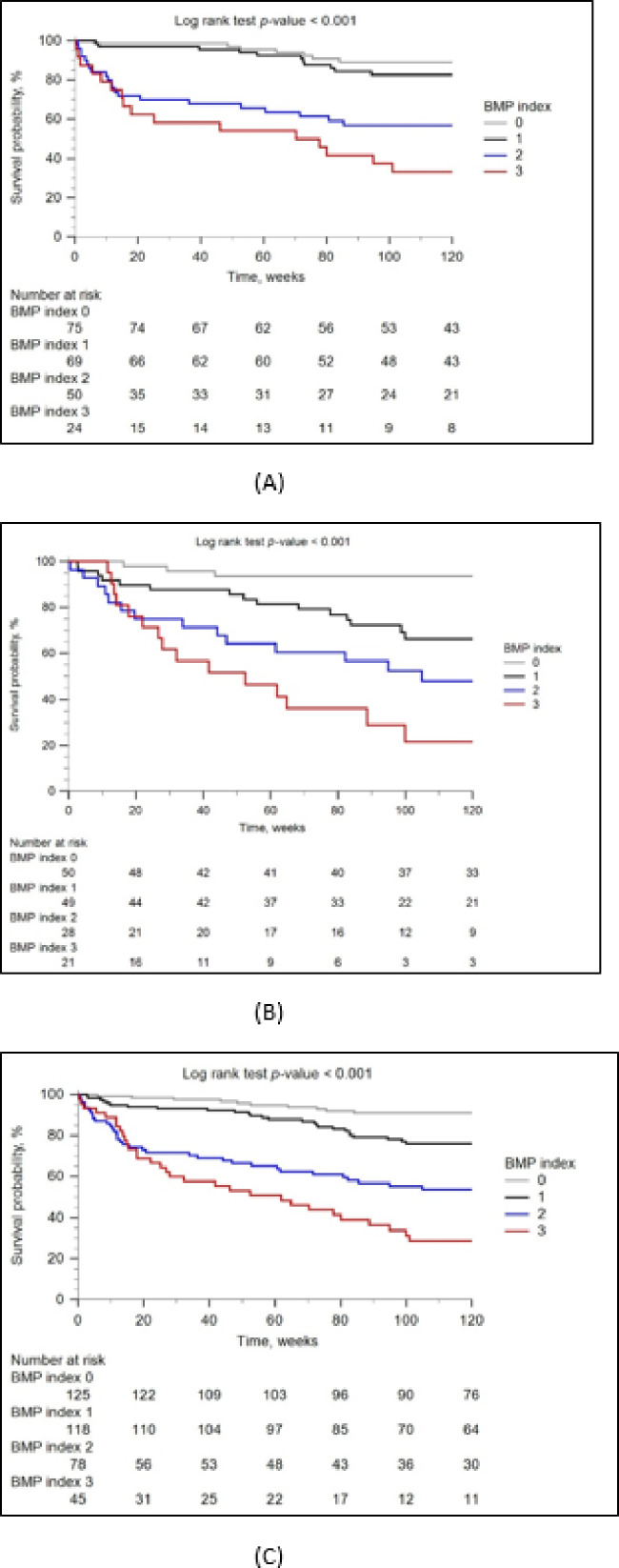
Kaplan–Meier curves illustrating the different probabilities of survival within 2 years of enrollment in the derivation cohort (**A**), validation cohort (**B**), and the combined whole cohort (**C**).

102 patients (28%) died within two years after diagnosis of fILDs (55 patients in the derivation and 47 in the validation cohort), with a median time of 30.6 weeks (IQR 10.9–71.6) between study enrollment and death. Most patients died from respiratory causes (Supplementary Table S7). Similar to the findings concerning AE, patients who died early were more likely to have, at baseline, a lower BMI, higher CCI, higher levels of mucin-1 and PTX3, higher probabilities of PH, poorer pulmonary functions, higher GAP stages, and higher BMP indices compared to patients who survived (Supplementary Table S8). In both derivation and validation cohorts, the proportions of patients who died early increased with the BMP index (Supplementary Table S3) regardless of immunosuppressant use before study enrollment (Supplementary Table S4) or subsequent administration of antifibrotic therapy (Supplementary Table S5). In Kaplan–Meier analyses, different BMP indices stratified patients with different cumulative survival rates within 2 years of enrollment (the time of fILD diagnosis; Fig. 3A,B,C). In univariate and multivariable Cox proportional hazard regression analyses, increasing BMP indices from index 1 indicated consecutively higher hazard ratios of early death in the derivation, validation, and combined whole cohorts (Table 3).

**Table 3:** Univariate and multivariable Cox proportional hazard regression analyses of the risk of early death in patients across the derivation, validation, and combined whole cohorts.

BMP index	Derivation cohort (*n* = 218)		Validation cohort (*n* = 148)		Whole cohort (*n* = 366)	
	Crude HR of early death^1^ (95% CI)	*P* value	Crude HR of early death^1^ (95% CI)	*P* value	Crude HR of early death^1^ (95% CI)	*P* value
0	Ref.	Ref.	Ref.	Ref.	Ref.	Ref.
1	1.69 (0.65–4.36)	0.28	5.73 (1.66–19.81)	0.006	2.86 (1.38–5.92)	0.005
2	5.74 (2.44–13.51)	< 0.001	10.89 (3.13–37.93)	< 0.001	7.26 (3.60–14.67)	< 0.001
3	9.97 (4.10–24.27)	< 0.001	19.71 (5.67–68.59)	< 0.001	12.90 (6.31–26.36)	< 0.001

BMP index	Adjusted HR of early death^2^ (95% CI)	*P* value	Adjusted HR of early death^2^ (95% CI)	*P* value	Adjusted HR of early death^2^ (95% CI)	*P* value
0	Ref.	Ref.	Ref.	Ref.	Ref.	Ref.
1	1.49 (0.58–3.87)	0.41	3.24 (0.93–11.35)	0.065	2.28 (1.09–4.74)	0.028
2	4.42 (1.85–10.54)	< 0.001	3.95 (1.09–14.31)	0.037	4.61 (2.25–9.46)	< 0.001
3	5.26 (2.09–13.29)	< 0.001	4.63 (1.27–16.87)	0.020	5.29 (2.52–11.11)	< 0.001

^1^Values were derived from univariate Cox proportional hazard regression analysis. ^2^Values were derived from multivariable Cox proportional hazard regression analysis, which adjusted for GAP stages, the Charlson comorbidity index, and echocardiographic probabilities of pulmonary hypertension. GAP, gender-age-physiology; HR, hazard ratio; Ref., the reference group in the determination of hazard ratios; 95% CI, 95% confidence interval.

### Comparative performance analyses

For predicting both AE and early death, univariate models using the complete BMP index consistently achieved the highest Td-AUCs and C-indices across all cohorts, outperforming models incorporating individual “B,” “M,” or “P” components. Supplementary Table S9 presents the distribution of patients experiencing AE and early death across distinct strata defined by nine previously reported predictors. Models involving the BMP index showed consistently higher Td-AUCs and C-indices than those based on monocyte counts, NLR, LMR, CPI, the CPB index, AISI, LIPI, or the HAL index. Compared to models using GAP staging for AE risk prediction, the BMP index exhibited comparable (as in the validation cohort) or slightly higher (as in the derivation and whole cohorts) Td-AUCs and C-indices. For early death prediction, models with the BMP index had Td-AUCs and C-indices that were comparable (as in the derivation cohort) or slightly lower (as in the validation and whole cohorts) to those using the GAP index but remained within a strong predictive range (Tables 5 and 6).

**Table 4:** Time-dependent areas-under-the-curves at median times to acute exacerbation and early death of various univariate predictive models.

Univariate Cox proportional hazard regression analysis for acute exacerbation			
Major predictor	Derivation cohort	Validation cohort	Whole cohort
BMI < or ≥ 24	0.63 (0.54–0.72)	0.67 (0.57–0.76)	0.64 (0.57–0.70)
Mucin-1 < or ≥ 2.5	0.82 (0.75–0.90)	0.74 (0.64–0.84)	0.78 (0.72–0.84)
PTX3 < or ≥ 2.2	0.81 (0.74–0.88)	0.77 (0.68–0.87)	0.80 (0.74–0.85)
Monocyte	0.64 (0.55–0.74)	0.52 (0.41–0.64)	0.57 (0.50–0.65)
NLR < or ≥ 2.19	0.68 (0.63–0.73)	0.61 (0.53–0.69)	0.65 (0.61–0.70)
LMR < or ≥ 4.18	0.52 (0.45–0.59)	0.51 (0.43–0.58)	0.49 (0.44–0.54)
AISI < or ≥ 434	0.66 (0.58–0.75)	0.63 (0.53–0.73)	0.64 (0.58–0.71)
LIPI 0 or 1–2	0.63 (0.55–0.70)	0.59 (0.49–0.70)	0.61 (0.55–0.68)
HAL groups	0.59 (0.50–0.67)	0.55 (0.45–0.66)	0.57 (0.50–0.64)
CPI ^1^ ≤ or > 41	0.74 (0.66–0.82)	0.83 (0.79–0.88)	0.78 (0.73–0.83)
CPB stages	0.71 (0.64–0.77)	0.72 (0.64–0.80)	0.71 (0.66–0.76)
GAP stages	0.79 (0.70–0.88)	0.84 (0.78–0.90)	0.80 (0.74–0.86)
BMP index	0.89 (0.83–0.95)	0.84 (0.75–0.92)	0.86 (0.81–0.91)
Univariate Cox proportional hazard regression analysis for early death			
Major predictor	Derivation cohort	Validation cohort	Whole cohort
BMI < or ≥ 24	0.59 (0.49–0.68)	0.61 (0.50–0.71)	0.60 (0.53–0.67)
Mucin-1 < or ≥ 2.5	0.74 (0.64–0.83)	0.68 (0.57–0.79)	0.71 (0.65–0.78)
PTX3 < or ≥ 2.2	0.79 (0.71–0.86)	0.67 (0.56–0.78)	0.73 (0.67–0.80)
Monocyte	0.62 (0.53–0.72)	0.53 (0.41–0.64)	0.56 (0.48–0.63)
NLR < or ≥ 2.19	0.65 (0.59–0.71)	0.55 (0.46–0.65)	0.61 (0.56–0.66)
LMR < or ≥ 4.18	0.49 (0.42–0.56)	0.53 (0.46–0.59)	0.51 (0.46–0.56)
AISI < or ≥ 434	0.65 (0.56–0.74)	0.63 (0.52–0.73)	0.64 (0.54–0.71)
LIPI 0 or 1–2	0.66 (0.59–0.73)	0.60 (0.50–0.71)	0.63 (0.57–0.69)
HAL groups	0.60 (0.51–0.69)	0.59 (0.49–0.69)	0.59 (0.53–0.66)
CPI ^1^ ≤ or > 41	0.64 (0.52–0.77)	0.73 (0.61–0.86)	0.68 (0.59–0.77)
CPB stages	0.69 (0.62–0.76)	0.67 (0.56–0.78)	0.68 (0.62–0.74)
GAP stages	0.77 (0.68–0.85)	0.84 (0.78–0.90)	0.80 (0.75–0.86)
BMP index	0.83 (0.76–0.90)	0.74 (0.63–0.84)	0.79 (0.73–0.85)

The time-dependent areas-under-the-curves were calculated at median times to the major adverse events, using R (version 4.4.3) and the packages ”*survival*” and “*timeROC*”. Values in parenthesis indicate the 95% confidence intervals. The median time (in weeks) to acute exacerbation of each cohort: derivation 14.7; validation 23.1; whole 21.5. The median time (in weeks) to early death of each cohort: derivation 25.1; validation 32.1; whole 30.6. Please refer to the main text for the full forms and references of the predictors.

^1^Sixty-one patients lacked the data of diffusion capacity for carbon monoxide due to intolerance to the test. Therefore only 305 patients (186 in the derivation cohort and 119 in the validation cohort) were included for analyses regarding CPI.

**Table 5:** Harrell’s C-indices of various univariate predictive models assessing risks of acute exacerbation and early death.

Univariate Cox proportional hazard regression analysis for acute exacerbation			
Major predictor	Derivation cohort	Validation cohort	Whole cohort
BMI < or ≥ 24	0.58 (0.52–0.65)	0.64 (0.58–0.71)	0.61 (0.56–0.65)
Mucin-1 < or ≥ 2.5	0.74 (0.69–0.80)	0.74 (0.67–0.80)	0.74 (0.70–0.79)
PTX3 < or ≥ 2.2	0.75 (0.70–0.80)	0.72 (0.65–0.78)	0.73 (0.69–0.77)
Monocyte	0.59 (0.51–0.66)	0.52 (0.45–0.60)	0.54 (0.49–0.59)
NLR < or ≥ 2.19	0.64 (0.59–0.68)	0.61 (0.55–0.67)	0.63 (0.59–0.66)
LMR < or ≥ 4.18	0.51 (0.46–0.56)	0.54 (0.49–0.58)	0.51 (0.48–0.54)
AISI < or ≥ 434	0.60 (0.54–0.66)	0.62 (0.55–0.69)	0.61 (0.56–0.65)
LIPI 0 or 1–2	0.61 (0.56–0.67)	0.61 (0.54–0.69)	0.61 (0.57–0.66)
HAL groups	0.60 (0.55–0.67)	0.57 (0.50–0.64)	0.59 (0.55–0.64)
CPI ^1^ ≤ or > 41	0.69 (0.62–0.75)	0.76 (0.70–0.83)	0.72 (0.67–0.76)
CPB stages	0.64 (0.58–0.69)	0.67 (0.61–0.73)	0.65 (0.61–0.69)
GAP stages	0.72 (0.66–0.79)	0.79 (0.75–0.84)	0.75 (0.71–0.79)
BMP index	**0.79 (0.73–0.85)**	**0.80 (0.74–0.86)**	**0.80 (0.76–0.83)**
Univariate Cox proportional hazard regression analysis for early death			
Major predictor	Derivation cohort	Validation cohort	Whole cohort
BMI < or ≥ 24	0.59 (0.53–0.66)	0.66 (0.60–0.73)	0.63 (0.58–0.67)
Mucin-1 < or ≥ 2.5	0.67 (0.60–0.73)	0.66 (0.59–0.73)	0.67 (0.62–0.71)
PTX3 < or ≥ 2.2	0.68 (0.62–0.74)	0.61 (0.54–0.68)	0.65 (0.60–0.70)
Monocyte	0.58 (0.51–0.64)	0.53 (0.46–0.61)	0.53 (0.48–0.58)
NLR < or ≥ 2.19	0.63 (0.58–0.68)	0.59 (0.53–0.66)	0.62 (0.58–0.65)
LMR < or ≥ 4.18	0.52 (0.47–0.56)	0.53 (0.49–0.58)	0.52 (0.49–0.55)
AISI < or ≥ 434	0.60 (0.54–0.66)	0.58 (0.51–0.65)	0.59 (0.55–0.64)
LIPI 0 or 1–2	0.59 (0.53–0.65)	0.60 (0.54–0.67)	0.60 (0.55–0.64)
HAL groups	0.60 (0.54–0.66)	0.62 (0.55–0.68)	0.61 (0.57–0.65)
CPI ^1^ ≤ or > 41	0.63 (0.55–0.71)	0.71 (0.63–0.79)	0.66 (0.60–0.72)
CPB stages	0.63 (0.57–0.68)	0.66 (0.59–0.73)	0.64 (0.60–0.68)
GAP stages	0.73 (0.67–0.79)	0.80 (0.76–0.85)	0.76 (0.72–0.80)
BMP index	**0.73 (0.67–0.79)**	**0.73 (0.67–0.80)**	**0.73 (0.69–0.78)**

Harrell’s C-indices were calculated using R (version 4.4.3) and the packages ”***survival***” and “***prodlim***”. Values in parenthesis indicate the 95% confidence intervals. Please refer to the main text for the full forms and references of the predictors.

^1^Sixty-one patients lacked the data of diffusion capacity for carbon monoxide due to intolerance to the test. Therefore only 305 patients (186 in the derivation cohort and 119 in the validation cohort) were included for analyses regarding CPI.

### Sensitivity analyses

We assessed the prognostic performance of the BMP index in the aforementioned pooled subgroups: subgroup “IPF” (235 patients) versus subgroup “Non-IPF” (131 patients), and subgroup “AntiF” (183 patients) versus subgroup “No-AntiF” (183 patients). The proportion of patients experiencing either AE or early death increased consistently across all subgroups as BMP index values rose (Supplementary Table S10). For the prediction of AE, the BMP index stratified patients with consecutively increasing subdistribution hazard ratios in all subgroups (Supplementary Table S11). For the prediction of early death, the BMP index stratified patients according to different risks in the subgroups “Non-IPF” and “AntiF”, with consecutively higher indices indicating higher risks. In the subgroups “IPF” and “No-AntiF”, indices 2 and 3 indicated higher risks than that of indices 0 and 1, though the adjusted hazard ratios did not increase from index 2 to 3 (Supplementary Table S12). Additionally, when the cutoff for the “B” component was changed from 24 to 25 kg/m^2^, the BMP index demonstrated comparable performance in predicting AE and early death risks, except in the validation cohort, the adjusted hazard ratio for early death associated with index 3 was not higher than that of index 2 (Supplementary Tables S13–S15). Furthermore, despite a hypothetically unidentified confounder with varying prevalence across all three cohorts, all multivariable analyses produced consistent results, supporting the robustness of our findings (Supplementary Figs. S2–S4).

## Discussion

In this study, we proposed and validated the novel BMP index for predicting severe adverse outcomes in patients with fILDs. The BMP index differentiated risks of AE and early death in all the study cohorts, with higher indices indicating higher risks. Each of the three components of the BMP index represents distinct but relevant pathophysiological mechanisms in fILDs. This multi-dimensional approach adds to its biological plausibility.

Compared to nine previously reported categorical prognostic predictors (including monocyte count, NLR, LMR, CPI, CPB index, HAL index, AISI, LIPI, and GAP staging^28–37^) the BMP index demonstrated superior or comparable predictive performance in terms of Td-AUCs and C-indices. Notably, for AE prediction, it outperformed all others, including GAP staging, which remains the most widely used prognostic indicator in clinical practice. Moreover, the BMP index circumvents the limitations of pulmonary function testing and radiation exposure associated with CT imaging, making it radiation-free and advantageous particularly for patients unable to undergo spirometry or D_LCO_ measurements.

Previous studies have identified BMI as a predictor of mortality risk in fILDs^13,15,17–20^, a finding supported by our present study. However, its prognostic role in AE remains uncertain. Yoon et al. reported that underweight IPF patients faced a higher risk of respiratory and all-cause hospitalization^18^. Among AE patients, Awano et al. found the highest in-hospital mortality risk in underweight individuals and the lowest in obese patients^15^. Other studies, however, reported no significant association between BMI and AE^20,38^. In our study, which included both IPF and non-IPF fILDs, BMI was inversely associated with AE occurrence (Supplementary Table S3), supporting the potential link between lower BMI and an increased risk of AE. Moreover, we adopted 24 kg/m^2^ as the BMI cutoff for scoring the “B” component, aligning with Taiwan’s officially established upper normal limit. Nevertheless, sensitivity analysis using 25 kg/m^2^ (which was commonly applied in previous studies conducted in European populations) yielded comparable results, supporting the feasibility of using this general WHO standard for the BMP index.

The present study has limitations. The influence of unidentified confounders (including the potential modifying effects of immunosuppressive and antifibrotic therapies) could not be entirely excluded, despite substantial efforts to mitigate this, such as comprehensive data collection, strict statistical control of confounders, validation using a distinct cohort, and sensitivity analyses of the major findings. Quantification of mucin-1 and PTX3 requires only a small blood volume and relies on commercially available and relatively inexpensive sandwich ELISA methods. These biomarkers, however, are not routinely measured in current clinical practice. Additionally, all participants were from hospitals in southern Taiwan. Biomarker levels, such as mucin-1 and PTX3, may be influenced by ethnic and genetic factors, including single nucleotide polymorphisms^39,40^, which were not assessed in this study. Although we demonstrated that the BMP index effectively stratifies the risk of AE and early death in the pooled subgroup “Non-IPF”, its predictive performance across distinct individual non-IPF ILD subtypes remains to be elucidated. Therefore, further prospective research, ideally involving a large and ethnically diverse patient population with IPF as well as various non-IPF ILDs, is warranted to evaluate the applicability of the BMP index across different ethnic groups and to determine its performance in the context of various genetic backgrounds and ILD subtypes.

In summary, the BMP index offers a noninvasive, spirometry-independent, and biologically grounded prognostic tool that facilitates early risk stratification in fILD patients. It may be especially beneficial in clinical settings where lung function testing is not feasible, and its simplicity makes it suitable for routine or even remote application. Future studies incorporating genetic profiling and broader international cohorts are warranted to confirm and extend its clinical utility.

## Methods

### Study design and population

This observational study included patients with fILDs who had been diagnosed, treated and followed between January 1, 2017, and May 31, 2025, at one of the three participating hospitals in southern Taiwan: National Cheng Kung University Hospital (NCKUH, a medical center), Tainan Hospital (TH, a regional hospital), and Chest Hospital (CH, a district hospital). This study was conducted in accordance with the Declaration of Helsinki and Taiwan’s Human Subjects Research Act. The study protocol was jointly approved by the Institutional Review Board of NCKUH (B-ER-105-390; A-ER-109-321; A-ER-111-595; B-ER-113-004). To be eligible, patients were required to be ≥ 20 years old, have a diagnosis of fILD confirmed by multi-disciplinary assessment, and have completed follow-up at the recruiting hospitals.

### Data collection and patient randomization

At study enrollment, basic demographic data (including BMI), specific fILD diagnosis, Charlson Comorbidity Index (CCI), smoking status, probability of pulmonary hypertension (PH), pulmonary function measurements, antifibrotic therapy, immunosuppressive treatments (for non-IPF fILDs), and gender-age-physiology (GAP) stages were recorded for each patient. Since all participants were followed at one of the three study hospitals, details of any subsequent major adverse events (including hospitalization, lung transplantation, and death) were also documented. During each follow-up visit, patients were carefully evaluated, and their electronic medical records, including those from the cloud-based medical records of National Health Insurance System (which covers over 99.5% of Taiwan’s population^41^, were regularly reviewed to update data on relevant treatments and adverse events, including those occurring at other medical institutions. We performed stratified randomization based on sex, types of fILDs (IPF or non-IPF), and GAP stages, to allocate all included patients into either a derivation cohort or a validation cohort in a 3:2 ratio.

### Important definitions

In this study, fILDs included both IPF and non-IPF fILDs. IPF was diagnosed according to international guidelines^3,42^. We defined a patient as having non-IPF fILD if the patient exhibited fibrosing features (traction bronchiectasis and/or honeycombing), in addition to the underlying ILD patterns, on HRCT images^1,3,8^. Acute exacerbation of fILDs was defined based on the 2016 international working group report, specifically excluding events with identifiable etiologies^9,43^. Early death referred to all-cause mortality within 2 years after diagnosis of fILDs. Fibrosing ILDs are typically associated with reduced survival, though heterogeneity exists. Patients who die within two years of diagnosis represent a high-risk group warranting heightened clinical attention. The probabilities of PH were determined based on transthoracic echocardiographic findings according to the 2022 ESC/ERS Guidelines^44^.

### Measurement of plasma mucin-1 and PTX3 levels

Baseline plasma levels of mucin-1 and PTX3 were measured at the time of study enrollment. After obtaining formal informed consent, an 8 mL blood specimen was collected from the patient using an EDTA vacuum collection tube. The specimen was immediately ice-bathed, centrifuged (at 4 °C and 1500 x g for 20 min using an Eppendorf Centrifuge 5810R), and the plasma extracted within 4 h under sterile conditions. We used the sandwich enzyme-linked immunosorbent assay (ELISA) kit (product number EH0406) from Fine Test (Wuhan Fine Biotech, Wuhan City, China) to measure mucin-1 levels, which employed antibodies targeting the tandem-repeat domain of the extracellular N-subunit of mucin-1. For measuring PTX3 levels, we used the ELISA kit (product number #E20031001) from Leadgene Biomedical, Inc.; Tainan, Taiwan, involving antibodies targeting the C-subunit of PTX3^25,45^. We performed all ELISAs in triplets according to manufactures’ protocols, and intra- and inter-assay coefficients of variation of < 15% were considered acceptable. All experiments were conducted at the Centre for Clinical Medical Research, NCKUH.

### Structure of the “BMP index” and cutoff values for scoring

Our proposed BMP index comprises three components. For the “B” component (baseline BMI), Taiwan’s officially established upper normal limit, modified from the WHO recommendation and set at 24 kg/m², has been adopted as the cutoff value^46^. For the “M” component (baseline plasma mucin-1 levels), a cutoff value of 2.5 ng/mL has been adopted from our previous studies involving patients with IPF^26,27^. For the “P” component (baseline plasma PTX3 levels), a cutoff value of 2.2 ng/mL has been adopted also from our previous work^25^. In practice, a score of 1 is assigned to each of the three components if meeting the following conditions: BMI < 24 kg/m^2^, mucin-1 level ≥ 2.5 ng/mL, and PTX3 level ≥ 2.2 ng/mL. A score of 0 is assigned otherwise. The BMP index is the sum of these scores, ranging from 0 to 3.

### Statistical analysis

We present categorical variables as counts and percentages, and continuous variables as means (standard deviation) if normally distributed or medians (interquartile range [IQR]) if not normally distributed. We did not impute missing values. We used Fischer’s exact test or the Mann–Whitney U test, whichever was appropriate, to test for non-random between-group differences in variables. We conducted Kaplan–Meier analysis with the log-rank test to stratify the univariate effect of BMP index on cumulative probabilities of AE and survival. The relationship between BMP index and the risk of AE over time was further analysed using multivariable Fine-Gray subdistribution regression, adjusting for GAP stages (a categorical quantification of age, sex, forced vital capacity and D_LCO_), CCI, probabilities of PH, and competing risks of death. The risk of early death among patients with different BMP indices were analysed using multivariable Cox proportional hazards regression, adjusting for GAP stages, CCI, and probabilities of PH. There was no multicollinearity among the variables. The Schoenfeld test was conducted to verify the proportional hazards assumption. The performance of the BMP index in the derivation cohort was independently verified in the validation cohort, and assessed in the pooled whole cohort. We analysed time-dependent areas-under-the-curve (Td-AUC) at median times to adverse events and Harrell’s C-index (C-index) for univariate predictive models across all cohorts. The Td-AUC and C-index of the BMP index were then compared with several recently reported categorical prognostic predictors, including GAP stages^28^, monocyte counts^29^, neutrophil to lymphocyte ratio (NLR)^30^, lymphocyte to monocyte ratio (LMR)^31^, the composite physiologic index (CPI)^32,33^, the clinical physiological biomarker (CPB) index^34^, the aggregate index of systemic inflammation (AISI)^35^, the lung immune prognostic index (LIPI)^36^, and the honeycomb-age-LDH (HAL) index^37^. Sensitivity analyses were conducted in three stages. First, we evaluated the prognostic performance of the BMP index across subgroups within the pooled whole cohort: patients with IPF (subgroup “IPF”) versus non-IPF fILDs (subgroup “Non-IPF”), and those receiving antifibrotic therapy (nintedanib or pirfenidone; subgroup “AntiF”) versus those not receiving (subgroup “No-AntiF”). Second, we tested the BMP index using a 25 kg/m^2^ cutoff (frequently employed in previous studies) for the “B” component, instead of the original 24 kg/m^2^ cutoff. Thirdly, we examined the potential influence of unidentified confounders on the observed associations. All statistical tests were two-tailed, and *p* < 0.05 was considered statistically significant. Statistical analyses were performed using R (version 4.4.3; The R Foundation for Statistical Computing, Vienna, Austria; https://www.r-project.org/) and MedCalc (version 22.0.2; MedCalc Software Ltd, Ostend, Belgium).

## Supplementary Information

Below is the link to the electronic supplementary material.

Supplementary Material 1

## Data Availability

The de-identified datasets used and analysed in this study are available from the corresponding authors upon reasonable request.
