# Supplementary material for: A novel, non-spirometric “BMP index” for predicting severe adverse outcomes in fibrosing interstitial lung diseases
Source: Sci Rep. 2025 Dec 14;16:2805. doi: 10.1038/s41598-025-32619-1 (PMC12824404; doi:10.1038/s41598-025-32619-1)
Supplement: Supplementary file 1 — Supplementary Material 1 [file 41598_2025_32619_MOESM1_ESM.pdf]

Title: A Novel, Non-Spirometric “BMP Index” for Predicting Severe Adverse Outcomes in Fibrosing Interstitial Lung Diseases

Authors: Tang-Hsiu Huang, Hsin-Yu Hou, Han-Yu Chang, Chia-Hao Hu, Hung-I Kuo, Hong-Ping Er, Yu-Wei Wu, Chien-Yu Lin, Yau-Lin Tseng, Ju-Ming Wang, Li-Ting Huang, Chia-Tse Weng, Sheng-Hsiang Lin, Chi-Chang Shieh*, Chao-Liang Wu*

(*Corresponding authors)

Supplementary Material

Supplementary Table S1: Diagnoses of underlying interstitial lung diseases

Diagnoses	Derivation cohort (n = 218)	Validation cohort (n = 148)
Idiopathic pulmonary fibrosis (IPF)	141 (65)	94 (64)
Idiopathic nonspecific interstitial pneumonia (iNSIP)	10 (5)	9 (6)
CTD-ILD with nonspecific interstitial pneumonia (NSIP) pattern	41 (19)	26 (17)
CTD-ILD with usual interstitial pneumonia (UIP) pattern	19 (9)	15 (10)
Fibrotic hypersensitivity pneumonia (fHP)	1 (0.5)	0 (0)
Unclassifiable interstitial lung diseases (ucILD)	5 (2)	3 (2)
Sarcoidosis, stage 4	0 (0)	1 (1)
Pleuroparenchymal fibroelastosis (PPFE)	1 (0.5)	0 (0)

Values indicate patient numbers and, in parentheses, % of the cohort total.

CTD, connective tissue diseases; ILD, interstitial lung diseases.

Supplementary Table S2: Baseline characteristics and early death rates of patients with and without acute exacerbation

Baseline characteristics	Derivation cohort (n = 218)		Validation cohort (n = 148)	
	Without AE (n = 158)	With AE (n = 60)	Without AE (n = 100)	With AE (n = 48)
Age, years	71.2 (63.5–77.8)	72.1 (64.1–80.5)	71.7 (64.4–79.0)	73.4 (67.3–84.0)
Sex Female, n (%)	45 (28)	11 (18)	26 (26)	14 (29)
Male, n (%)	113 (72)	49 (82)	74 (74)	34 (71)
Body mass index, kg/m^2	24.0 ± 3.5*	22.9 ± 4.5*	24.4 ± 3.4*	21.9 ± 4.2*
Cigarette smoking status				
Never smoker, n (%)	76 (48)	27 (45)	39 (39)	26 (54)
Current smoker, n (%)	17 (11)	2 (3)	13 (13)	2 (4)
Former smoker, n (%)	65 (41)	31 (52)	48 (48)	20 (42)
Charlson comorbidity index	4 (3–6)*	6 (3–7)*	5 (3–6)	5 (4–6)
Pulmonary hypertension probability[1]				
Low, n (%)	110 (70)*	29 (48)*	70 (70)*	21 (44)*
Intermediate, n (%)	37 (23)*	19 (32)*	18 (18)*	14 (29)*
High, n (%)	11 (7)*	12 (20)*	12 (12)*	13 (27)*
Diagnosis IPF	101 (64)	40 (67)	61 (61)	33 (69)
Non-IPF fILDs	57 (36)	20 (33)	39 (39)	15 (31)
Already initiated immunosuppressant[2], n (%)	52 (33)	17 (28)	29 (29)	14 (29)
Received antifibrotics during the study period, n (%)	80 (50)	32 (53)	40 (40)	31 (64)
Nintedanib, n (%)	41 (26)	19 (32)	15 (15)	14 (29)
Pirfenidone, n (%)	29 (18)	6 (10)	20 (20)	14 (29)
Ever switched between the two agents, n (%)[3]	10 (6)	7 (11)	5 (5)	3 (6)
FVC, L	2.28 ± 0.76*	1.91 ± 0.68*	2.46 ± 0.86*	1.71 ± 0.67*
FVC, % prediction	76 ±19*	62 ± 20*	82 ± 23*	58 ± 19*
D_{LCO}, mL/min/mmHg	10.4 (8.0–13.7)*	6.4 (5.1–11.2)*	11.2 (8.4–14.8)*	6.3 (5.2–9.6)*
D_{LCO}, % prediction	61 (50–83)*	42 (31–63)*	72 (55–86)*	43 (34–56)*
Mucin-1, ng/mL	0.70 (0.42–1.31)*	3.42 (1.66–6.39)*	0.80 (0.36–1.52)*	3.47 (1.93–5.86)*

(Supplementary Table S2 continues)

Baseline characteristics	Derivation cohort (n = 218)		Validation cohort (n = 148)	
	Without AE (n = 158)	With AE (n = 60)	Without AE (n = 100)	With AE (n = 48)
**PTX3, ng/mL**	1.22 (0.81–1.95)*	2.73 (1.79–5.24)*	1.05 (0.72–1.84)*	2.53 (1.86–4.66)*
**Stages based on the GAP index**				
**Stage 1, n (%)**	82 (52)*	16 (27)*	62 (62)*	4 (8)*
**Stage 2, n (%)**	61 (39)*	16 (27)*	28 (28)*	23 (48)*
**Stage 3, n (%)**	15 (9)*	28 (46)*	10 (10)*	21 (44)*
**BMP index 0, n (%)**	67 (43)*	8 (13)*	47 (47)*	3 (6)*
**1, n (%)**	62 (39)*	7 (12)*	38 (38)*	11 (23)*
**2, n (%)**	27 (17)*	23 (38)*	15 (15)*	13 (27)*
**3, n (%)**	2 (1)*	22 (37)*	0 (0)*	21 (44)*
**Early death, n (%)**	20 (13)*	35 (58)*	14 (14)*	33 (69)*

Categorical data are presented as counts and percentages, and continuous variables are presented as means (±standard deviation) if normally distributed or medians (interquartile range) if non-normally distributed. [1] The classification is based on 2022 ESC/ERS Guidelines for the diagnosis and treatment of pulmonary hypertension [reference 44]. [2] Immunosuppressants (referring to systemic corticosteroids and non-steroid immunosuppressants) were administered to patients with non-IPF fILDs. [3] These patients transitioned between the two antifibrotic agents due to intolerance to adverse effects. **Abbreviations**: AE, acute exacerbation; BMP, body mass index, mucin-1, pentraxin 3; D_{LCO}, diffusion capacity for carbon monoxide; fILD, fibrosing interstitial lung diseases; FVC, forced vital capacity; GAP, gender-age-physiology; IPF, idiopathic pulmonary fibrosis; PTX3, pentraxin 3. * *P*-value < 0.05 for the comparison between patients with and those without AE.

Supplementary Table S3: Proportions of patients with and without major adverse events in strata according to individual components and total scores of the BMP index.

Components of the BMP index	Derivation cohort (n = 218)				Validation cohort (n = 148)				Total patient numbers in the strata
	Acute exacerbation		Early death		Acute exacerbation		Early death		
	No (n = 158)	Yes (n = 60)	No (n = 163)	Yes (n = 55)	No (n = 100)	Yes (n = 48)	No (n = 101)	Yes (n = 47)	
**BMI, kg/m²**									
**Underweight (<18.5)[1]**	11 (58)*	8 (42)*	9 (47)**	10 (53)**	3 (25)**	9 (75)**	3 (25)**	9 (75)**	31
**Normal (18.5–23.9)[1]**	63 (68)*	30 (32)*	65 (70)**	28 (30)**	37 (59)**	26 (41)**	34 (54)**	29 (46)**	156
**Overweight (≥ 24)[1]**	84 (79)*	22 (21)*	89 (84)**	17 (16)**	60 (82)**	13 (18)**	64 (88)**	9 (12)**	179
**BMI, kg/m²**									
**< 24**	74 (66)*	38 (34)*	74 (66)**	38 (34)**	40 (53)**	35 (47)**	37 (49)**	38 (51)**	187
**≥ 24**	84 (79)*	22 (21)*	89 (84)**	17 (16)**	60 (82)**	13 (18)**	64 (88)**	9 (12)**	179
**Mucin-1, ng/mL**									
**< 2.5**	141 (87)**	22 (13)**	137 (84)**	26 (16)**	89 (86)**	14 (14)**	83 (81)**	20 (19)**	266
**≥ 2.5**	17 (31)**	38 (69)**	26 (47)**	29 (53)**	11 (24)**	34 (76)**	18 (40)**	27 (60)**	100
**PTX3, ng/mL**									
**< 2.2**	127 (88)**	17 (12)**	123 (85)**	21 (15)**	83 (83)**	17 (17)**	76 (76)*	24 (24)*	244
**≥ 2.2**	31 (42)**	43 (58)**	40 (54)**	34 (46)**	17 (35)**	31 (65)**	25 (52)*	23 (48)*	122
**BMP index total score**									
**0**	67 (89)**	8 (11)**	68 (90)**	7 (10)**	47 (94)**	3 (6)**	47 (94)**	3 (6)**	125
**1**	62 (90)**	7 (10)**	58 (84)**	11 (16)**	38 (78)**	11 (22)**	34 (69)**	15 (31)**	118
**2**	27 (54)**	23 (46)**	29 (58)**	21 (42)**	15 (54)**	13 (46)**	14 (50)**	14 (50)**	78
**3**	2 (8)**	22 (92)**	8 (33)**	16 (67)**	0 (0)**	21 (100)**	6 (29)**	15 (71)**	45

Values indicate patient numbers and % of the subgroup in parentheses. [1] These BMI criteria were issued by Taiwan's Health Promotion Administration, Ministry of Health and Welfare, which were modified from the recommendations of the World Health Organization. **Abbreviations**: BMI, body mass index; BMP, body mass index, mucin-1, pentraxin 3; PTX3, pentraxin 3.

* *P*-value < 0.05 by Fischer's exact test. ** *P*-value < 0.005 by Fischer's exact test.

Supplementary Table S4: Status of immunosuppressive therapy at study enrollment, baseline BMP index scoring, and distribution of major adverse events in patients with non-IPF fibrosing interstitial lung diseases.

Baseline characteristics	Patients without immunosuppressive therapy before study enrollment (n = 20)				Patients already initiated immunosuppressive therapy before study enrollment (n = 111)			
**BMI, kg/m^2**	23.5 ± 4.8				23.4 ± 4.2			
**Plasma mucin-1 level, ng/mL**	1.07 (0.58 – 1.70)				1.22 (0.62 – 2.72)			
**Plasma PTX3 level, ng/mL**	1.35 (0.77 – 3.12)				1.87 (1.01 – 3.55)			
**BMP index total score:**	**No AE** (n = 16)	**With AE** (n = 4)	**Survived** (n = 15)	**Early death** (n = 5)	**No AE** (n = 80)	**With AE** (n = 31)	**Survived** (n = 83)	**Early death** (n = 28)
**0**	8 (100)*	0 (0)*	8 (100)*	0 (0)*	25 (90)*	3 (10)*	25 (89)*	3 (11)*
**1**	5 (100)*	0 (0)*	5 (100)*	0 (0)*	37 (88)*	5 (12)*	35 (83)*	7 (17)*
**2**	3 (50)*	3 (50)*	2 (33)*	4 (67)*	17 (68)*	8 (32)*	18 (72)*	7 (28)*
**3**	0 (0)*	1 (100)*	0 (0)*	1 (100)*	1 (6)*	15 (94)*	5 (31)*	11 (69)*

Values of BMI (normally distributed) are presented as mean ± standard deviation; values of mucin-1 and PTX3 (both not normally distributed) are presented as median (interquartile ranges). There was ***no*** statistically significant difference between the groups in baseline BMI and plasma levels of mucin-1 and PTX3. For the distribution of adverse events across different scores of the BMP index, values indicate patient numbers and % of the subgroup in parentheses. **Abbreviations**: AE, acute exacerbation; BMI, body mass index; BMP, body mass index, mucin-1, pentraxin 3; IPF, idiopathic pulmonary fibrosis; PTX3, pentraxin 3. * *P*-value < 0.005 by Fischer's exact test.

Supplementary Table S5: Status of antifibrotic therapy during the study period, baseline BMP index scoring, and distribution of major adverse events.

Baseline characteristics	Patients without antifibrotic therapy during the study period (n = 183)				Patients receiving antifibrotic therapy during the study period (n = 183)			
**BMI, kg/m²**	23.4 ± 4.0				23.9 ± 3.6			
**Plasma mucin-1 level, ng/mL**	0.91 (0.49 – 2.17)				1.27 (0.56 – 2.80)			
**Plasma PTX3 level, ng/mL**	1.53 (0.87 – 3.20)				1.42 (0.90 – 2.42)			
**BMP index total score:**	**No AE** (n = 138)	**With AE** (n = 45)	**Survived** (n =131)	**Early death** (n = 52)	**No AE** (n = 120)	**With AE** (n = 63)	**Survived** (n = 133)	**Early death** (n = 50)
**0**	60 (95)*	3 (5)*	58 (92)*	5 (8)*	54 (87)*	8 (13)*	57 (92)*	5 (8)*
**1**	52 (93)*	4 (7)*	43 (77)*	13 (23)*	48 (77)*	14 (23)*	49 (79)*	13 (21)*
**2**	24 (67)*	18 (43)*	23 (55)*	19 (45)*	18 (50)*	18 (50)*	20 (56)*	16 (44)*
**3**	2 (9)*	20 (91)*	7 (32)*	15 (68)*	0 (0)*	23 (100)*	7 (30)*	16 (70)*

Values of BMI (normally distributed) are presented as mean ± standard deviation; values of mucin-1 and PTX3 (both not normally distributed) are presented as median (interquartile ranges). There was ***no*** statistically significant difference between the groups in baseline BMI and plasma levels of mucin-1 and PTX3. For the distribution of adverse events across different scores of the BMP index, values indicate patient numbers and % of the subgroup in parentheses. **Abbreviations**: AE, acute exacerbation; BMI, body mass index; BMP, body mass index, mucin-1, pentraxin 3; IPF, idiopathic pulmonary fibrosis; PTX3, pentraxin 3. * *P*-value < 0.005 by Fischer's exact test

Supplementary Table S6 Univariate and multivariable Fine-Gray subdistribution regression analyses of the risk of acute exacerbation within 2 years of enrollment in patients across the derivation, validation, and combined whole cohorts.

BMP index	Derivation cohort (n = 218)		Validation cohort (n = 148)		Whole cohort (n = 366)	
	**Crude sdHR of AE within 2 years[1] (95% CI)**	***P* value**	**Crude sdHR of AE within 2 years[1] (95% CI)**	***P* value**	**Crude sdHR of AE within 2 years[1] (95% CI)**	***P* value**
**0**	Ref.	Ref.	Ref.	Ref.	Ref.	Ref.
**1**	1.29 (0.40–4.14)	0.66	4.23 (0.89–20.00)	0.069	2.15 (0.87–5.27)	0.096
**2**	6.15 (2.30–16.46)	< 0.001	14.53 (3.28–64.50)	< 0.001	8.53 (3.76–19.34)	< 0.001
**3**	23.08 (8.79–60.62)	< 0.001	45.96 (10.69 – 197.60)	< 0.001	29.79 (13.54–65.58)	< 0.001
**BMP index**	**Adjusted sdHR of AE within 2 years[2] (95% CI)**	***P* value**	**Adjusted sdHR of AE within 2 years[2] (95% CI)**	***P* value**	**Adjusted sdHR of AE within 2 years[2] (95% CI)**	***P* value**
**0**	Ref.	Ref.	Ref.	Ref.	Ref.	Ref.
**1**	1.04 (0.32–3.37)	0.95	2.76 (0.58–13.08)	0.20	1.68 (0.68 – 4.17)	0.26
**2**	4.50 (1.68–12.07)	0.003	6.58 (1.37–31.57)	0.019	5.42 (2.34 – 12.55)	< 0.001
**3**	12.30 (4.63–32.71)	< 0.001	17.51 (3.97–77.19)	< 0.001	13.71 (6.01 – 31.25)	< 0.001

[1] Values were derived from univariate Fine-Gray subdistribution hazard regression analysis controlling for the competing risk of death.

[2] Values were derived from multivariable Fine-Gray subdistribution hazard regression analysis, which accounted for the competing risk of death and also adjusted for GAP stages, the Charlson comorbidity index, and echocardiographic probabilities of pulmonary hypertension. **Abbreviations:** AE, acute exacerbation; GAP, gender-age-physiology; Ref., the reference group in the determination of subdistribution hazard ratios; sdHR, subdistribution hazard ratio; 95% CI, 95% confidence interval.

Supplementary Table S7: Causes of early death

Derivation cohort		Validation cohort	
**Causes of early death**	**Numbers (%)**	**Causes of early death**	**Numbers (%)**
Acute exacerbation	17 (31)	Acute exacerbation	17 (36)
Infectious pneumonia	13 (23)	Progression of fibrosing interstitial lung diseases	17 (36)
Progression of fibrosing interstitial lung diseases	10 (18)	Infectious pneumonia	6 (13)
Acute coronary syndrome / cardiac failure	4 (7)	Acute coronary syndrome / cardiac failure	3 (7)
Lung cancer	3 (5)	Urinary tract infection with septic shock	2 (4)
Out of hospital cardiac arrest (OHCA)	2 (4)	Biliary tract infection and septic shock	1 (2)
Urinary tract infection with septic shock	2 (4)	Lung cancer	1 (2)
Massive gastrointestinal hemorrhage	2 (4)		
Flare of chronic hepatitis B	1 (2)		
Hemorrhagic stroke	1 (2)		
**Total number of early death**	**55**	**Total number of early death**	**47**

Numbers in parentheses indicate percentages of each cohort’s total number of early deaths

Supplementary Table S8: Baseline characteristics and acute exacerbation rates of patients who died within two years versus those who survived.

Baseline characteristics	Derivation cohort (n = 218)		Validation cohort (n = 148)	
	Alive > 2 years (n = 163)	Died within 2 years (n = 55)	Alive > 2 years (n = 101)	Died within 2 years (n = 47)
Age, years	70.4 (62.8–76.3)*	75.3 (65.5–84.1)*	70.2 (63.4–77.7)*	73.6 (70.0–84.0)*
Sex Female, n (%)	46 (28)	10 (18)	25 (25)	15 (32)
Male, n (%)	117 (72)	45 (82)	76 (75)	32 (68)
Body mass index, kg/m^2	24.3 ± 3.6*	22.1 ± 4.0*	24.4 ± 3.3*	21.7 ± 4.3*
Cigarette smoking status				
Never smoker, n (%)	82 (50)	21 (38)	42 (42)	23 (49)
Current smoker, n (%)	16 (10)	3 (6)	14 (13)	1 (2)
Former smoker, n (%)	65 (40)	31 (56)	45 (45)	23 (49)
Charlson comorbidity index	4 (3–6)*	6 (4–8)*	4 (3–6)*	5 (4–6)*
Pulmonary hypertension probability[1]				
Low, n (%)	105 (64)	34 (62)	72 (71)*	19 (40)*
Intermediate, n (%)	44 (27)	12 (22)	17 (17)*	15 (32)*
High, n (%)	14 (9)	9 (16)	12 (12)*	13 (28)*
Diagnosis IPF	103 (63)	38 (69)	63 (62)	31 (66)
Non-IPF fILDs	60 (37)	17 (31)	38 (38)	16 (34)
Already initiated immunosuppressant[2], n (%)	55 (34)	14 (25)	29 (29)	14 (30)
Received antifibrotics during the study period, n (%)	87 (53)	25 (45)	46 (46)	25 (53)
Nintedanib, n (%)	46 (28)	14 (25)	18 (18)	11 (23)
Pirfenidone, n (%)	26 (16)	9 (16)	21 (21)	13 (28)
Ever switched between the two agents, n (%)[3]	15 (9)	2 (4)	7 (7)	1 (2)
FVC, L	2.29 ± 0.73*	1.86 ± 0.76*	2.49 ± 0.78*	1.64 ± 0.79*
FVC, % prediction	76 ±19*	61 ± 20*	83 ± 21*	57 ± 22*
D_{LCO}, mL/min/mmHg	10.4 (7.8–13.6)*	7.2 (5.7–9.6)*	11.2 (8.3–14.7)*	6.6 (5.2–9.6)*
D_{LCO}, % prediction	61 (45–83)*	50 (35–60)*	71 (55–86)*	48 (34–58)*
Mucin-1, ng/mL	0.74 (0.46–1.47)*	2.88 (1.33–4.92)*	0.84 (0.38–1.67)*	2.60 (1.39–4.54)*

(Supplementary Table S8 continues)

Baseline characteristics	Derivation cohort (n = 218)		Validation cohort (n = 148)	
	Alive > 2 years (n = 163)	Died within 2 years (n = 55)	Alive > 2 years (n = 101)	Died within 2 years (n = 47)
**PTX3, ng/mL**	1.22 (0.82–2.16)*	2.40 (1.44–3.62)*	1.25 (0.72–2.16)*	2.04 (1.31–4.25)*
**Stages based on the GAP index**				
**Stage 1, n (%)**	89 (55)*	9 (17)*	64 (63)*	2 (4)*
**Stage 2, n (%)**	57 (35)*	20 (36)*	30 (30)*	21 (45)*
**Stage 3, n (%)**	17 (10)*	26 (47)*	7 (7)*	24 (51)*
**BMP index 0, n (%)**	68 (42)*	7 (13)*	47 (46)*	3 (6)*
**1, n (%)**	58 (35)*	11 (20)*	34 (34)*	15 (32)*
**2, n (%)**	29 (18)*	21 (38)*	14 (14)*	14 (30)*
**3, n (%)**	8 (5)*	16 (29)*	6 (6)*	15 (32)*
**Acute exacerbation, n (%)**	25 (15)*	35 (64)*	15 (15)*	33 (70)*

Categorical data are presented as counts and percentages, and continuous variables are presented as means (±standard deviation) if normally distributed or medians (interquartile range) if non-normally distributed. [1] The classification is based on 2022 ESC/ERS Guidelines for the diagnosis and treatment of pulmonary hypertension [reference 44]. [2] Immunosuppressants (referring to systemic corticosteroids and non-steroid immunosuppressants) were administered to patients with non-IPF fILDs. [3] These patients transitioned between the two antifibrotic agents due to intolerance to adverse effects. **Abbreviations**: BMP, body mass index, mucin-1, pentraxin 3; D_{LCO}, diffusion capacity for carbon monoxide; fILD, fibrosing interstitial lung diseases; FVC, forced vital capacity; GAP, gender-age-physiology; IPF, idiopathic pulmonary fibrosis; PTX3, pentraxin 3.

* *P*-value < 0.05 for the comparison between patients who died within 2 years and those who survived.

Supplementary Table S9: Proportions of patients with and without major adverse events in strata according to various previously reported predictors

Predictors and cutoff values / stages		Derivation cohort (n = 218)				Validation cohort (n = 148)			
		Acute exacerbation		Early death		Acute exacerbation		Early death	
		No (n = 158)	Yes (n = 60)	No (n = 163)	Yes (n = 55)	No (n = 100)	Yes (n = 48)	No (n = 101)	Yes (n = 47)
**Monocyte**	**< 0.6 x 10^9 cells/L**	65 (78)*	18 (22)*	68 (82)	15 (18)	45 (67)	22 (33)	44 (66)	23 (34)
	**0.6 ~ 0.95 x 10^9 cells/L**	74 (72)*	27 (28)*	73 (72)	28 (28)	37 (67)	18 (33)	37 (67)	18 (33)
	**≥ 0.95 x 10^9 cells/L**	19 (56)*	15 (44)*	22 (65)	12 (35)	18 (69)	8 (31)	20 (77)	6 (23)
**NLR**	**< 2.19**	65 (88)*	9 (12)*	67 (91)*	7 (9)*	39 (87)*	6 (13)*	39 (87)*	6 (13)*
	**≥ 2.19**	93 (65)*	51 (35)*	96 (67)*	48 (33)*	61 (59)*	42 (41)*	62 (60)*	41 (40)*
**LMR**	**≥ 4.18**	19 (68)	9 (32)	23 (82)	5 (18)	17 (85)	3 (15)	16 (80)	4 (20)
	**< 4.18**	139 (73)	51 (27)	140 (74)	50 (26)	83 (65)	45 (35)	85 (66)	43 (34)
**CPI** [1]	**≤ 41**	86 (88)[1]*	9 (12)[1]*	86 (88)[1]*	9 (12)[1]*	66 (93)[1]*	5 (7)[1]*	65 (92)[1]*	6 (8)[1]*
	**> 41**	60 (66)[1]*	31 (34)[1]*	68 (75)[1]*	23 (25)[1]*	26 (54)[1]*	22 (46)[1]*	28 (58)[1]*	20 (42)[1]*
**AISI**	**< 434**	88 (78)	25 (22)	93 (82)*	20 (18)*	60 (80)*	15 (20)*	57 (76)	18 (24)
	**≥ 434**	70 (67)	35 (33)	70 (67)*	35 (33)*	40 (55)*	33 (45)*	44 (60)	29 (40)
**LIPI**	**Score = 0**	70 (83)*	14 (17)*	70 (83)*	14 (17)*	54 (82)*	12 (18)*	53 (80)*	13 (20)*
	**Score ≥ 0**	88 (66)*	46 (34)*	93 (69)*	41 (31)*	46 (56)*	36 (44)*	48 (59)*	34 (41)*
**HAL**	**Score 0**	23 (100)*	0 (0)*	22 (96)*	1 (4)*	8 (89)	1 (11)	8 (89)*	1 (11)*
	**Score 1**	58 (74)*	20 (26)*	62 (79)*	16 (21)*	35 (76)	11 (24)	38 (83)*	8 (17)*
	**Score 2/3**	77 (66)*	40 (34)*	79 (68)*	38 (32)*	57 (61)	36 (39)	55 (59)*	38 (41)*
**CPB**	**Stage 1**	35 (90)*	4 (10)*	38 (97)*	1 (3)*	33 (87)*	5 (13)*	32 (84)*	6 (16)*
	**Stage 2**	50 (75)*	17 (25)*	49 (73)*	18 (27)*	32 (74)*	11 (26)*	35 (81)*	8 (19)*
	**Stage 3**	73 (65)*	39 (35)*	76 (68)*	36 (32)*	35 (52)*	32 (48)*	34 (51)*	33 (49)*

(Supplementary Table S9 continues on the next page.)

(Supplementary Table S9 continues.)

Predictors and cutoff values / stages		Derivation cohort (n = 218)				Validation cohort (n = 148)			
		Acute exacerbation		Early death		Acute exacerbation		Early death	
		No (n = 158)	Yes (n = 60)	No (n = 163)	Yes (n = 55)	No (n = 100)	Yes (n = 48)	No (n = 101)	Yes (n = 47)
**GAP**	**Stage 1**	82 (84)*	16 (16)*	89 (91)*	9 (9)*	62 (94)*	4 (6)*	64 (97)*	2 (3)*
	**Stage 2**	61 (79)*	16 (21)*	57 (74)*	20 (26)*	28 (55)*	23 (45)*	30 (59)*	21 (41)*
	**Stage 3**	15 (35)*	28 (65)*	17 (40)*	26 (60)*	10 (32)*	21 (68)*	7 (23)*	24 (77)*

Values indicate patient numbers and % of the subgroup in parentheses. Please refer to the main text for the references of the predictors. **Abbreviations:** AISI, aggregate index of systemic inflammation; CPB, clinical physiological biomarker; CPI, composite physiologic index; GAP, gender-age-physiology; HAL, honeycomb, age, lactate dehydrogenase; LIPI, lung immune prognostic index; LMR, lymphocyte to monocyte ratio; NLR, neutrophil to lymphocyte ratio.

[1] Sixty-one patients lacked the data of diffusion capacity for carbon monoxide due to intolerance to the test. Therefore only 305 patients (186 in the derivation cohort and 119 in the validation cohort) were included for analyses regarding CPI.

* *P*-value < 0.05 by Fischer's exact test.

Supplementary Table S10: Proportions of patients in each pooled subgroups, stratified by total scores of the BMP index, with and without the major adverse events.

BMP index	Subgroup "IPF" (n = 235)		Subgroup "Non-IPF" (n = 131)		Subgroup "AntiF" (n = 183)		Subgroup "No-AntiF" (n = 183)	
	**Without AE**	**With AE**	**Without AE**	**With AE**	**Without AE**	**With AE**	**Without AE**	**With AE**
**0**	81 (91)**	8 (9)**	33 (92)**	3 (8)**	54 (87)**	8 (13)**	60 (95)**	3 (5)**
**1**	58 (82)**	13 (18)**	42 (89)**	5 (11)**	48 (77)**	14 (23)**	52 (93)**	4 (7)**
**2**	22 (47)**	25 (53)**	20 (65)**	11 (35)**	18 (50)**	18 (50)**	24 (57)**	18 (43)**
**3**	1 (4)**	27 (96)**	1 (6)**	16 (94)**	0 (0)**	23 (100)**	2 (9)**	20 (91)**
	**Survived**	**Early death**	**Survived**	**Early death**	**Survived**	**Early death**	**Survived**	**Early death**
**0**	82 (92)**	7 (8)**	33 (92)**	3 (8)**	57 (92)**	5 (8)**	58 (92)**	5 (8)**
**1**	52 (73)**	19 (27)**	40 (85)**	7 (15)**	49 (79)**	13 (21)**	43 (77)**	13 (23)**
**2**	23 (49)**	24 (51)**	20 (65)**	11 (35)**	20 (56)**	16 (44)**	23 (55)**	19 (45)**
**3**	9 (32)**	19 (68)**	5 (29)**	12 (71)**	7 (30)**	16 (70)**	7 (32)**	15 (68)**

Values indicate patient numbers and % of the strata in parentheses. **The 4 subgroups:** "IPF", patients with idiopathic pulmonary fibrosis; "Non-IPF", patients with fibrosing interstitial lung diseases other than IPF; "AntiF", patients who received antifibrotic therapy; "No-AntiF", patients without any antifibrotic therapy. **Abbreviations**: AE, acute exacerbation.

* *P*-value < 0.05 by Fischer's exact test. ** *P*-value < 0.005 by Fischer's exact test.

Supplementary Table S11: Univariate and multivariable Fine-Gray subdistribution regression analyses of the risk of acute exacerbation in subgroups of the pooled whole cohort.

BMP index	Subgroup "IPF" (n = 235)		Subgroup "Non-IPF" (n = 131)		Subgroup "AntiF" (n = 183)		Subgroup "No-AntiF" (n = 183)	
	**Crude sdHR of AE[1] (95% CI)**	***P*-value**	**Crude sdHR of AE[1] (95% CI)**	***P*-value**	**Crude sdHR of AE[1] (95% CI)**	***P*-value**	**Crude sdHR of AE[1] (95% CI)**	***P*-value**
**0**	Ref.	Ref.	Ref.	Ref.	Ref.	Ref.	Ref.	Ref.
**1**	2.12 (0.89–2.03)	0.089	1.25 (0.30–5.18)	0.76	1.85 (0.79–4.34)	0.15	1.46 (0.32–6.54)	0.62
**2**	8.13 (3.66–18.06)	<0.001	4.79 (1.39–16.57)	0.013	5.03 (2.18–11.65)	<0.001	10.84 (3.25–36.23)	<0.001
**3**	24.97 (11.80–52.86)	<0.001	24.56 (8.58–88.53)	<0.001	19.44 (9.34–40.45)	<0.001	41.43 (12.59–136.35)	<0.001
**BMP index**	**Adjusted sdHR of AE[2] (95% CI)**	***P*-value**	**Adjusted sdHR of AE[2] (95% CI)**	***P*-value**	**Adjusted sdHR of AE[2] (95% CI)**	***P*-value**	**Adjusted sdHR of AE[2] (95% CI)**	***P*-value**
**0**	Ref.	Ref.	Ref.	Ref.	Ref.	Ref.	Ref.	Ref.
**1**	1.55 (0.63–3.81)	0.34	1.00 (0.23–4.30)	0.99	1.64 (0.69–3.89)	0.26	1.07 (0.24–4.89)	0.93
**2**	5.21 (2.27–11.96)	<0.001	2.88 (0.69–12.07)	0.15	4.64 (2.01–10.72)	<0.001	5.34 (1.49–19.11)	0.01
**3**	12.70 (5.85–27.56)	<0.001	17.40 (4.49–67.45)	<0.001	14.43 (6.81–30.61)	<0.001	16.19 (4.46–58.78)	<0.001

[1] Values were derived from univariate Fine-Gray subdistribution hazard regression analysis controlling for the competing risk of death.

[2] Values were derived from multivariable Fine-Gray subdistribution hazard regression analysis, which accounted for the competing risk of death and also adjusted for GAP stages, the Charlson comorbidity index, and echocardiographic probabilities of pulmonary hypertension. **The 4 subgroups:** "IPF", patients with idiopathic pulmonary fibrosis; "Non-IPF", patients with fibrosing interstitial lung diseases other than IPF; "AntiF", patients who received antifibrotic therapy; "No-AntiF", patients without any antifibrotic therapy. **Abbreviations:** AE, acute exacerbation; GAP, gender-age-physiology; Ref., the reference group in the determination of subdistribution hazard ratios; sdHR, subdistribution hazard ratio; 95% CI, 95% confidence interval.

Supplementary Table S12: Univariate and multivariable Cox proportional hazard regression analyses of the risk of early death in subgroups of the pooled whole cohort.

BMP index	Subgroup "IPF" (n = 235)		Subgroup "Non-IPF" (n = 131)		Subgroup "AntiF" (n = 183)		Subgroup "No-AntiF" (n = 183)	
	**Crude HR of early death[1] (95% CI)**	***P*-value**	**Crude HR of early death[1] (95% CI)**	***P*-value**	**Crude HR of early death[1] (95% CI)**	***P*-value**	**Crude HR of early death[1] (95% CI)**	***P*-value**
**0**	Ref.	Ref.	Ref.	Ref.	Ref.	Ref.	Ref.	Ref.
**1**	3.57 (1.50–8.49)	0.004	1.85 (0.48–7.16)	0.372	2.69 (0.96–7.56)	0.059	3.08 (1.10–8.63)	0.033
**2**	8.84 (3.81–20.53)	<0.001	5.03 (1.40–18.03)	0.013	7.26 (2.66–19.84)	<0.001	7.22 (2.69–19.35)	<0.001
**3**	12.94 (5.42–30.85)	<0.001	12.47 (3.51–44.30)	<0.001	13.53 (4.93–37.08)	<0.001	12.86 (4.67–35.47)	<0.001
**BMP index**	**Adjusted HR of early death[2] (95% CI)**	***P*-value**	**Adjusted HR of early death[2] (95% CI)**	***P*-value**	**Adjusted HR of early death[2] (95% CI)**	***P*-value**	**Adjusted HR of early death[2] (95% CI)**	***P*-value**
**0**	Ref.	Ref.	Ref.	Ref.	Ref.	Ref.	Ref.	Ref.
**1**	2.68 (1.12–6.41)	0.027	2.08 (0.51–8.49)	0.306	2.26 (0.80–6.38)	0.124	2.58 (0.91–7.31)	0.074
**2**	5.55 (2.34–13.17)	<0.001	4.04 (1.01–16.13)	0.048	5.69 (2.05–15.81)	<0.001	3.67 (1.31–10.32)	0.013
**3**	5.16 (2.06–12.93)	<0.001	7.81 (2.04–29.93)	0.003	9.29 (3.26–26.50)	<0.001	3.35 (1.17–9.62)	0.025

[1] Values were derived from univariate Cox proportional hazard regression analysis. [2] Values were derived from multivariable Cox proportional hazard regression analysis, which adjusted for GAP stages, the Charlson comorbidity index, and echocardiographic probabilities of pulmonary hypertension. **The 4 subgroups:** "IPF", patients with idiopathic pulmonary fibrosis; "Non-IPF", patients with fibrosing interstitial lung diseases other than IPF; "AntiF", patients who received antifibrotic therapy; "No-AntiF", patients without any antifibrotic therapy. **Abbreviations:** GAP, gender-age-physiology; HR, hazard ratio; Ref., the reference group in the determination of hazard ratios; 95% CI, 95% confidence interval.

Supplementary Table S13: Univariate and multivariable Fine-Gray subdistribution regression analyses of acute exacerbation risk across the derivation, validation, and whole cohorts, using 25 kg/m² cutoff for the "B" component of the BMP index.

B_{25}MP index[1]	Derivation cohort (n = 218)		Validation cohort (n = 148)		Whole cohort (n = 366)	
	**Crude sdHR of AE[2] (95% CI)**	***P*-value**	**Crude sdHR of AE[2] (95% CI)**	***P*-value**	**Crude sdHR of AE[2] (95% CI)**	***P*-value**
**0**	Ref.	Ref.	Ref.	Ref.	Ref.	Ref.
**1**	1.44 (0.46–4.56)	0.54	2.69 (0.58–12.40)	0.20	1.91 (0.77–4.72)	0.16
**2**	6.52 (2.25–18.90)	<0.001	7.82 (1.70–35.90)	0.008	7.00 (2.92–16.77)	<0.001
**3**	29.72 (10.20–86.63)	<0.001	39.19 (9.03–170.10)	<0.001	33.44 (14.27–78.38)	<0.001
**B_{25}MP index[1]**	**Adjusted sdHR of AE[3] (95% CI)**	***P*-value**	**Adjusted sdHR of AE[3] (95% CI)**	***P*-value**	**Adjusted sdHR of AE[3] (95% CI)**	***P*-value**
**0**	Ref.	Ref.	Ref.	Ref.	Ref.	Ref.
**1**	1.13 (0.34–3.77)	0.84	2.20 (0.47–10.32)	0.32	1.59 (0.63–3.98)	0.33
**2**	4.90 (1.64–14.60)	0.004	4.05 (0.81–20.19)	0.088	4.84 (1.97–11.93)	<0.001
**3**	17.21 (5.59–53.01)	<0.001	17.91 (3.75–85.66)	<0.001	17.84 (7.26–43.88)	<0.001

[1] A cutoff of 25 kg/m² was used for the "B" component, while the cutoff values for the other two components remained unchanged. [2]Values were derived from univariate Fine-Gray subdistribution hazard regression analysis controlling for the competing risk of death. [3] Values were derived from multivariable Fine-Gray subdistribution hazard regression analysis, which accounted for the competing risk of death and also adjusted for GAP stages, the Charlson comorbidity index, and echocardiographic probabilities of pulmonary hypertension. **Abbreviations:** AE, acute exacerbation; GAP, gender-age-physiology; Ref., the reference group in the determination of subdistribution hazard ratios; sdHR, subdistribution hazard ratio; 95% CI, 95% confidence interval.

Supplementary Table S14: Univariate and multivariable Cox proportional hazard regression analyses of early death risk across the derivation, validation, and whole cohorts, using 25 kg/m² cutoff for the "B" component of the BMP index.

B_{25}MP index[1]	Derivation cohort (n = 218)		Validation cohort (n = 148)		Whole cohort (n = 366)	
	**Crude HR of early death[2] (95% CI)**	***P*-value**	**Crude HR of early death[2] (95% CI)**	***P*-value**	**Crude HR of early death[2] (95% CI)**	***P*-value**
**0**	Ref.	Ref.	Ref.	Ref.	Ref.	Ref.
**1**	1.57 (0.56–4.41)	0.390	2.33 (0.67–8.13)	0.183	1.90 (0.86–4.18)	0.112
**2**	4.68 (1.75–12.54)	0.002	6.91 (2.00–23.89)	0.002	5.49 (2.54–11.86)	<0.001
**3**	10.73 (3.98–28.93)	<0.001	10.38 (2.99–36.07)	<0.001	10.65 (4.91–23.09)	<0.001
**B_{25}MP index[1]**	**Adjusted HR of early death[3] (95% CI)**	***P*-value**	**Adjusted HR of early death[3] (95% CI)**	***P*-value**	**Adjusted HR of early death[3] (95% CI)**	***P*-value**
**0**	Ref.	Ref.	Ref.	Ref.	Ref.	Ref.
**1**	1.37 (0.49–3.86)	0.551	1.54 (0.43–5.47)	0.504	1.54 (0.70–3.39)	0.289
**2**	3.45 (1.27–9.41)	0.015	2.64 (0.73–9.54)	0.139	3.34 (1.52–7.36)	0.003
**3**	5.45 (1.93–15.42)	0.001	2.46 (0.68–8.91)	0.171	4.18 (1.88–9.31)	<0.001

[1] A cutoff of 25 kg/m^2 was used for the "B" component, while the cutoff values for the other two components remained unchanged.

[2] Values were derived from univariate Cox proportional hazard regression analysis. [3] Values were derived from multivariable Cox proportional hazard regression analysis, which adjusted for GAP stages, the Charlson comorbidity index, and echocardiographic probabilities of pulmonary hypertension. **Abbreviations**: GAP, gender-age-physiology; HR, hazard ratio; Ref., the reference group in the determination of hazard ratios; 95% CI, 95% confidence interval.

Supplementary Table S15: Time-dependent area-under-the-curve (Td-AUC) and Harrell's C-index for predictive models assessing risks of acute exacerbation and early death, using a BMI threshold of 25 kg/m² to score the BMP index

**Univariate Cox proportional hazard regression analysis for acute exacerbation**			
	**Derivation**	**Validation**	**Whole**
**Td-AUC**	0.88 (0.82–0.95)	0.83 (0.74–0.92)	0.86 (0.80–0.91)
**Harrell's C-index**	0.80 (0.75–0.86)	0.79 (0.73–0.85)	0.80 (0.76–0.84)
**Univariate Cox proportional hazard regression analysis for early death**			
	**Derivation**	**Validation**	**Whole**
**Td-AUC**	0.83 (0.75–0.90)	0.72 (0.60–0.83)	0.78 (0.71–0.85)
**Harrell's C-index**	0.73 (0.66–0.79)	0.70 (0.63–0.77)	0.72 (0.67–0.76)

Values in parenthesis indicate the 95% confidence intervals. The time-dependent areas-under-curves (Td-AUC) were calculated at median times to the major adverse events, using R (version 4.4.3) and the packages "***survival***" and "***timeROC***". Harrell's C-indices were calculated using R (version 4.4.3) and the packages "***survival***" and "***prodlim***". The median time (in weeks) to acute exacerbation of each cohort: derivation 14.7; validation 23.1; whole 21.5. The median time (in weeks) to early death of each cohort: derivation 25.1; validation 32.1; whole 30.6.

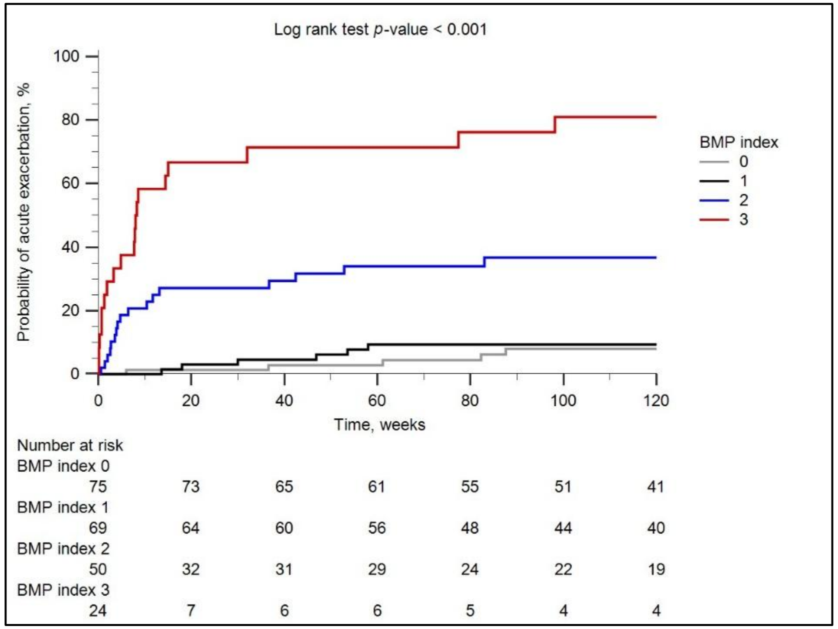

(1A)

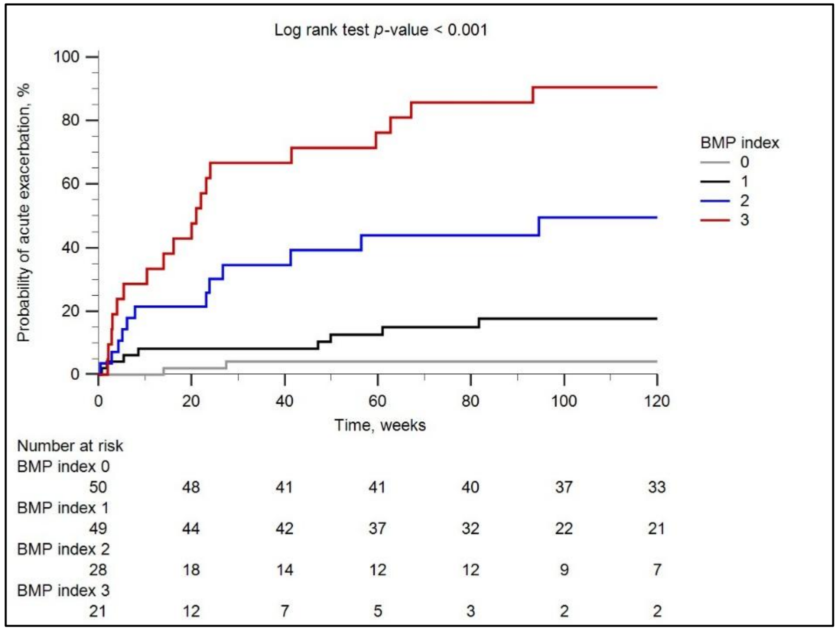

(1B)

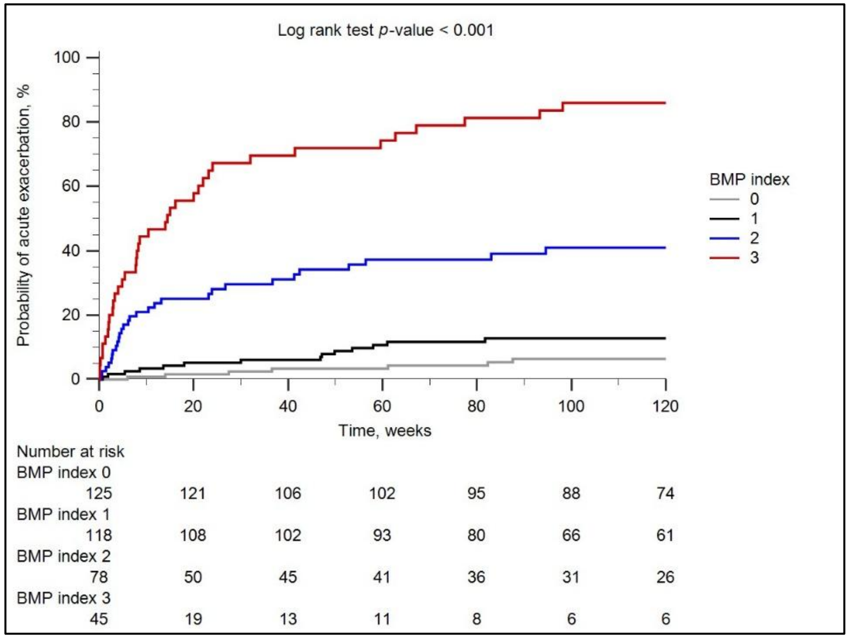

Supplementary Figure S1: Kaplan-Meier curves illustrating the cumulative probability of developing acute exacerbation within 2 years of enrollment in the derivation cohort (1A), validation cohort (1B), and the combined whole cohort (1C)

(1C)

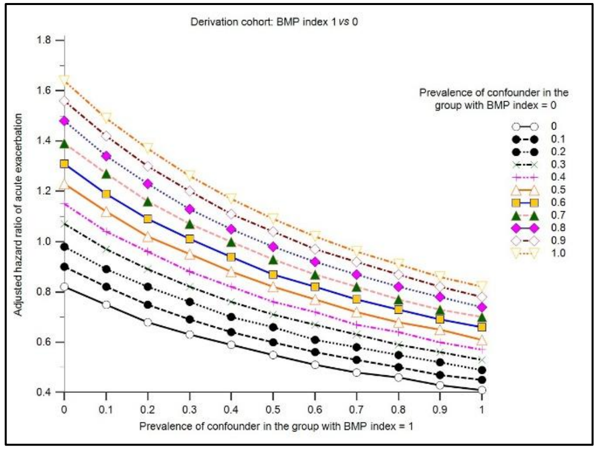

(2A)

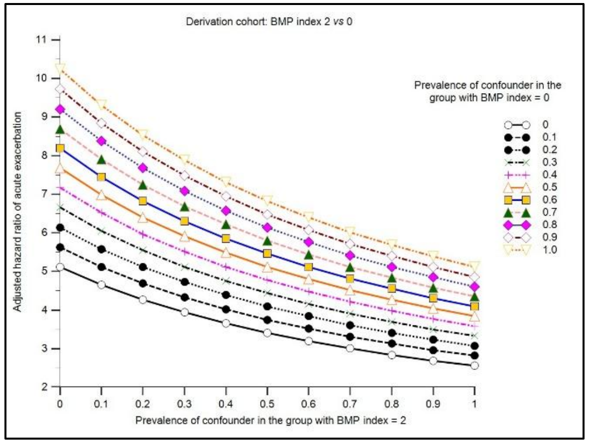

(2B)

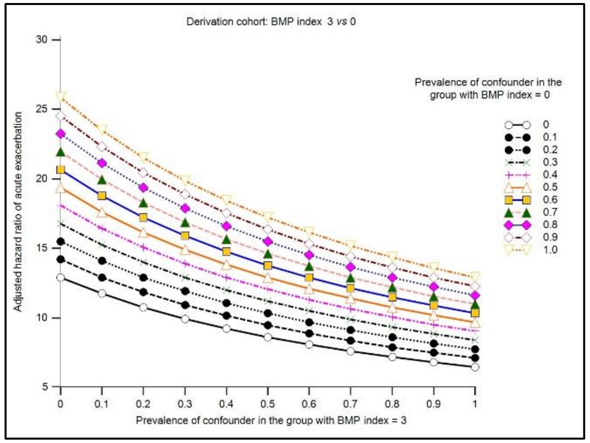

(2C)

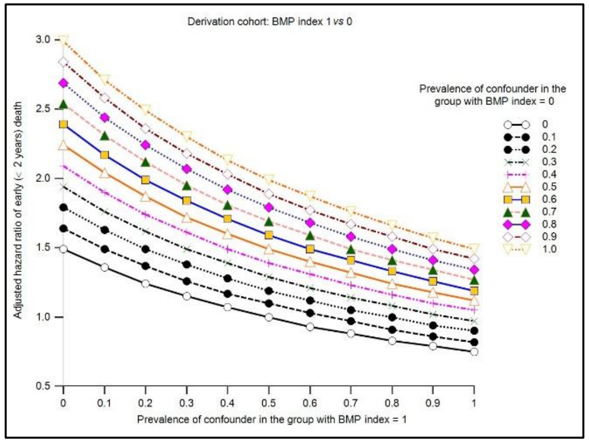

(2D)

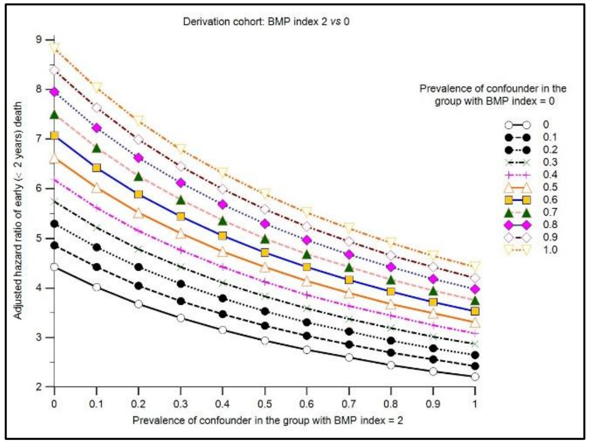

(2E)

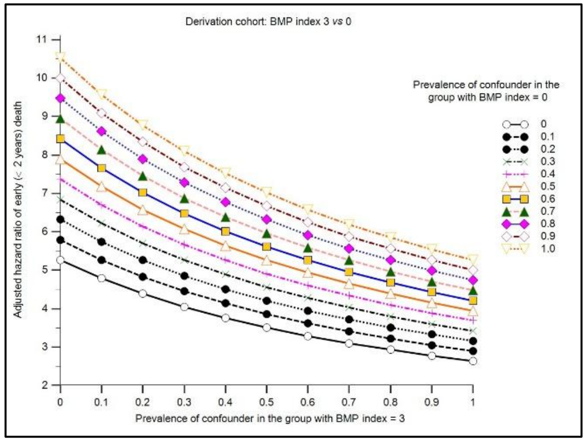

(2F)

Supplementary Figure S2: Results of sensitivity analyses showing the influence from an unidentified confounder of various prevalence in the ***derivation cohort*** on the relationship between adjusted hazard ratios (aHR) of acute exacerbation (AE) and: (2A) BMP index 1 *vs* 0; (2B) BMP index 2 *vs* 0; (2C) BMP index 3 *vs* 0, and on the relationship between aHR of early death and: (2D) BMP index 1 *vs* 0; (2E) BMP index 2 *vs* 0; (2F) BMP index 3 *vs* 0. For example, as in (2A), when hypothetically all patients with BMP index 0 have the unidentified confounder (the prevalence of this confounder was 1.0, as represented by the top orange broken line with hallow-triangular markers), but none of the patients with BMP index 1 have this unidentified confounder (thus the prevalence was 0), then BMP index 1 would still be a significant predictor of AE (aHR = 1.64). Analyses were performed using R (Version 4.4.3) and packages ***survival*** and ***obsSens***; graphs were plotted using MedCal (Version 20.118).

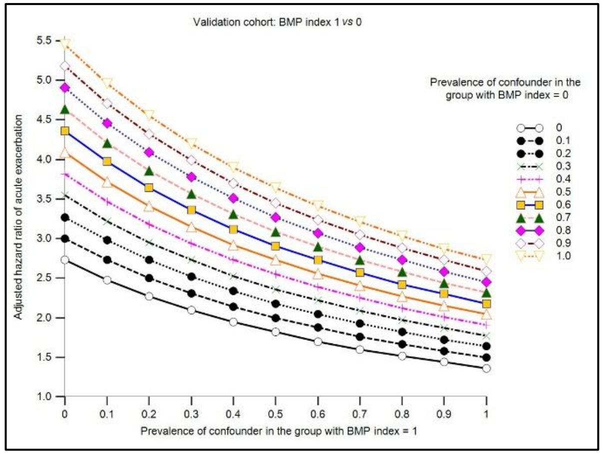

(3A)

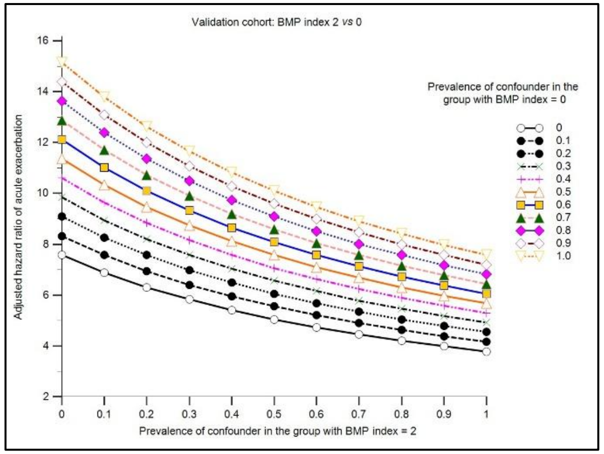

(3B)

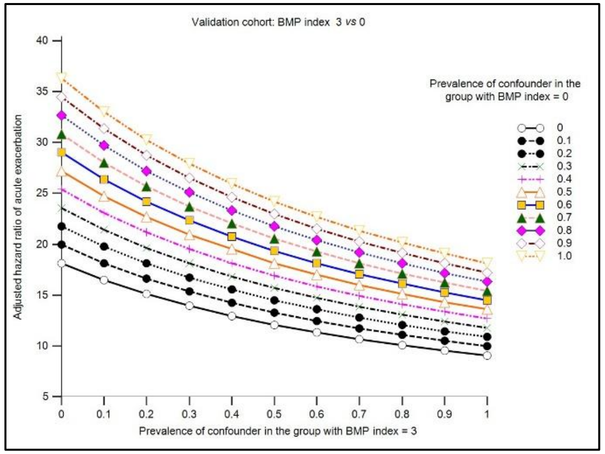

(3C)

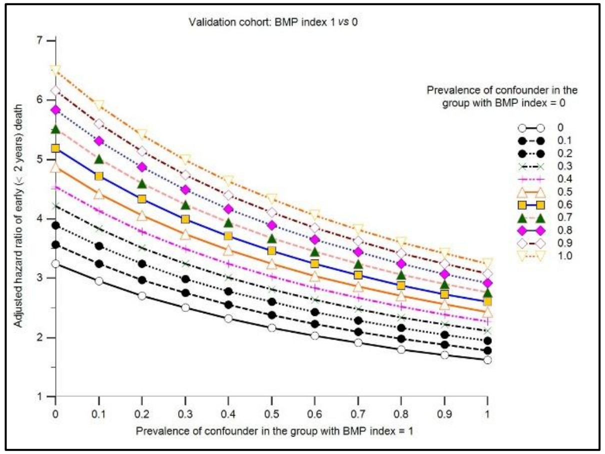

(3D)

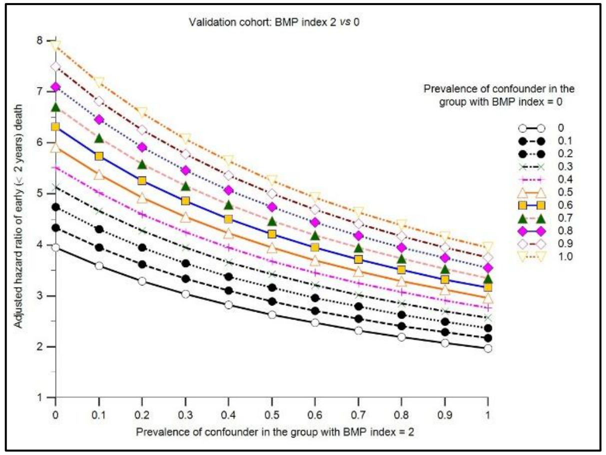

(3E)

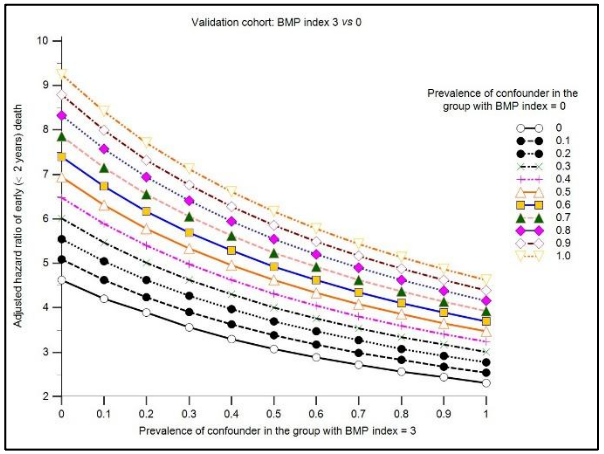

(3F)

Supplementary Figure S3: Results of sensitivity analyses showing the influence from an unidentified confounder of various prevalence in the ***validation cohort*** on the relationship between adjusted hazard ratios (aHR) of acute exacerbation (AE) and: (3A) BMP index 1 *vs* 0; (3B) BMP index 2 *vs* 0; (3C) BMP index 3 *vs* 0, and on the relationship between aHR of early death and: (3D) BMP index 1 *vs* 0; (3E) BMP index 2 *vs* 0; (3F) BMP index 3 *vs* 0. For example, as in (3A), when hypothetically all patients with BMP index 0 have the unidentified confounder (the prevalence of this confounder was 1.0, as represented by the top orange broken line with hallow-triangular markers), but none of the patients with BMP index 1 have this unidentified confounder (thus the prevalence was 0), then BMP index 1 would still be a significant predictor of AE (aHR = 5.45). Analyses were performed using R (Version 4.4.3) and packages ***survival*** and ***obsSens***; graphs were plotted using MedCal (Version 20.118).

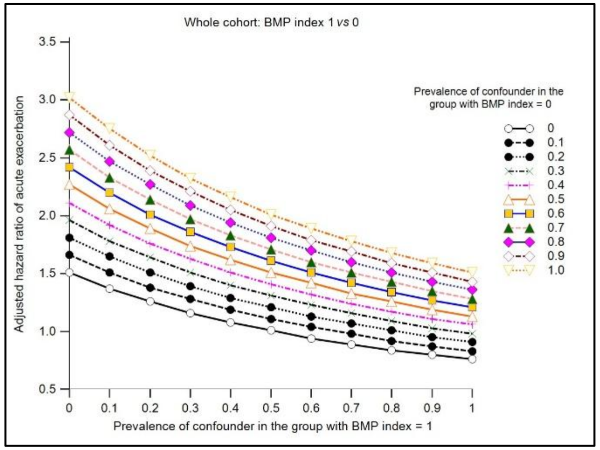

(4A)

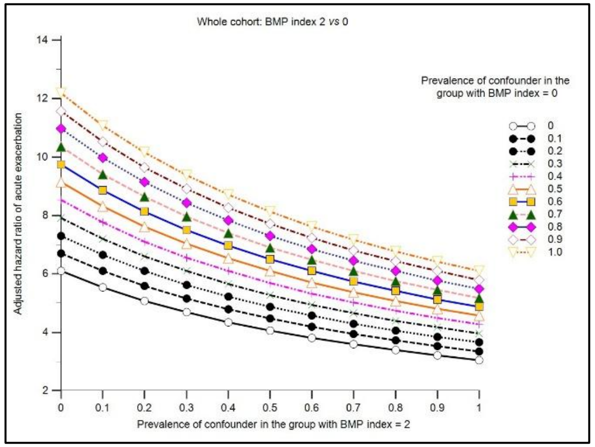

(4B)

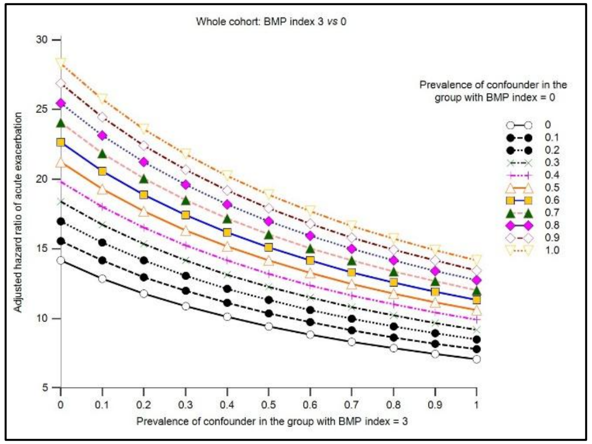

(4C)

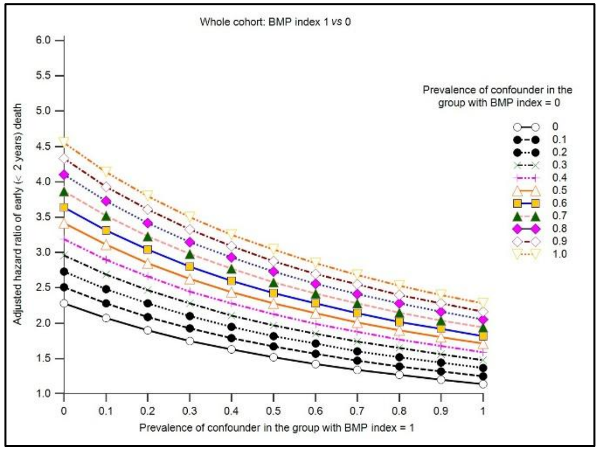

(4D)

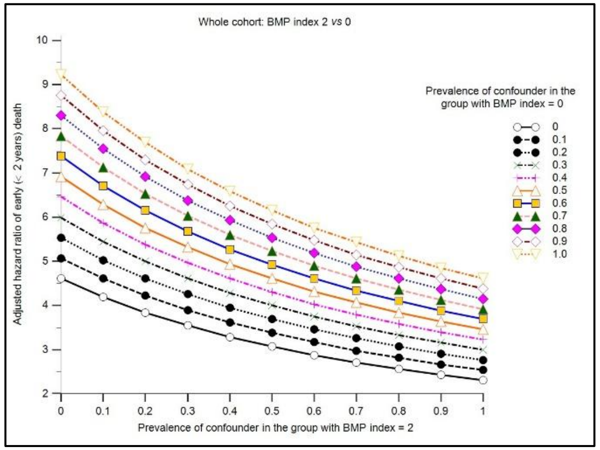

(4E)

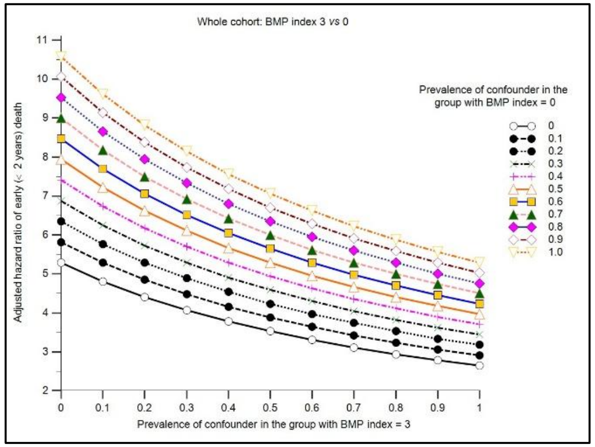

(4F)

Supplementary Figure S4: Results of sensitivity analyses showing the influence from an unidentified confounder of various prevalence in the pooled ***whole cohort*** on the relationship between adjusted hazard ratios (aHR) of acute exacerbation (AE) and: (4A) BMP index 1 *vs* 0; (4B) BMP index 2 *vs* 0; (4C) BMP index 3 *vs* 0, and on the relationship between aHR of early death and: (4D) BMP index 1 *vs* 0; (4E) BMP index 2 *vs* 0; (4F) BMP index 3 *vs* 0. For example, as in (4A), when hypothetically all patients with BMP index 0 have the unidentified confounder (the prevalence of this confounder was 1.0, as represented by the top orange broken line with hallow-triangular markers), but none of the patients with BMP index 1 have this unidentified confounder (thus the prevalence was 0), then BMP index 1 would still be a significant predictor of AE (aHR = 3.06). Analyses were performed using R (Version 4.4.3) and packages ***survival*** and ***obsSens***; graphs were plotted using MedCal (Version 20.118).
